
JOURNAL INFORMATION
==============================
NLM Title Abbreviation: J Orthop Traumatol
Journal ID: J Orthop Traumatol
NLM Journal ID: 101090931

Journal of Orthopaedics and Traumatology : Official Journal of the Italian Society of Orthopaedics and Traumatology

ISSN: 1590-9921
EISSN: 1590-9999
Publisher: Springer

ARTICLE INFORMATION
==============================
PMCID: PMC4417757
DOI: 10.1007/s10195-012-0209-8
Article ID: 209
Article version: 1
Subjects: Events

In-Depth Oral Presentations and Oral Communications

Electronic publication date: 2012 Aug 30
Print publication date: 2012 Nov
Volume: 13
Issue: Suppl 1
First page: 25
Last page: 55
Copyright: © The Author(s) 2012
License: Open AccessThis article is distributed under the terms of the Creative Commons Attribution 2.0 International License (https://creativecommons.org/licenses/by/2.0), which permits unrestricted use, distribution, and reproduction in any medium, provided the original work is properly cited.
License URL: https://creativecommons.org/licenses/by/2.0/

Keywords: Rotator Cuff, Anterior Cruciate Ligament Reconstruction, Radial Head, Soft Tissue Sarcoma, Adhesive Capsulitis
issue-copyright-statement: © The Author(s) 2012

==============================
J Orthopaed Traumatol (2012) 13 (Suppl 1):S25–S55
DOI 10.1007/s10195-012-0209-8

11 NOVEMBER 2012

### In-Depth Oral Presentations and Oral Communications

### IN-DEPTH ORAL PRESENTATIONS

### *AT01—PAINFUL PROSTHESES*

**Revision of knee prostheses with components malrotation: treatment algorithm using a computed tomographic analysis**

M. Fosco*[1], D. Tigani[2], E. Rimondi[3]

[1]Chirurgia di Revisione della Protesi d'Anca e Sviluppo Nuovi Impianti, Istituto Ortopedico Rizzoli (Bologna, IT);
[2]Dipartimento di Ortopedia, Ospedale Santa Maria alle Scotte (Siena, IT);
[3]Dipartimento di Radiologia Diagnostica ed Interventistica, Istituto Ortopedico Rizzoli (Bologna, IT)

**Introduction** Malrotation of prosthetic components have been widely recognised as a cause of knee prostheses failure. We hypothesised that an algorithm including clinical and radiographic evaluation, as well as a computed tomography analysis with defined values, could be reliable for the preoperative diagnosis of knee prosthesis malrotation in order to advise our treatment.
**Materials and methods** For this purpose, current parameters and related literature were taken into account. We analysed 44 patients with anterior knee pain after total knee arthroplasty. Among them, 24 patients (17 females and seven males), aged 43 to 78 years (mean 67 years) were evaluated with computed helicoidal tomography, and subsequently treated in a different way according to severity of their problem. Eight patients with components rotation in the normal range and patients with mild combined external rotation of the prosthesis underwent patella arthroplasty; other 16 patients with malposition in internal rotation or more severe external rotation, underwent complete prosthetic revision.
**Results** After an average follow-up of 32 months (range 12 to 120 months) results assessed according to the Knee Society rating system showed excellent or good clinical outcome in 87 % (21 out of 24 patients) and functionally in 75 % (18 out of 24 patients) of cases surgically treated.
**Discussion** The malposition of knee prosthetic components on the axial plane is an emerging problem, difficult to interpret, which provides various surgical options such as resurfacing of the patella in mild cases until the complete revision of the implant.
**Conclusions** We present an algorithm to detect exact cause of anterior knee pain after TKA, and that could help to choose appropriate surgical planning and treatment of these cases.

**Femoral overhang in the PS-TKA**

L. Limonciello*, L. Fantasia, F. Fantasia

(Foggia, IT)

**Introduction** During the implantation of a total knee arthroplasty (TKA) the posterior cruciate ligament (PCL) produces a flexion instability. To avoid this inconvenience the surgeons usually choose a larger femoral component but this option causes a mediolateral overhang. The aim of this study is establishing the overhang causes, particularly in posterior stabilized (PS) total knee arthroplasty.
**Materials and methods** We analysed 400 TKA performed by three surgeons. Two hundred were performed by PCL retaining (CR), and two hundred by PCL sacrificing. The study was performed on 162 females (81 %) and 38 males (19 %) treated by PS-TKA, and 152 (76 %) females and 48 (24 %) males treated by CR-TKA.
**Results** The femoral components in TKA were compared with the tibial ones. We obtained these results: about PS-TKA 81 (41 %) femoral components were larger, 94 (47 %) were identical, 25 (12 %) were smaller; about CR-TKA 26 (13 %) were larger, 102 (51 %) were identical, 72 (36 %) were smaller.
**Discussion** Several factors may produce a femoral component oversizing as incorrect positioning of anterior probe, excessive cutting block extrarotation. Anyway we focused on the different femoral components chosen in PS-TKA and CR-TKA. In our study we showed that when the surgeon sacrifices the PLC uses a larger femoral component.
**Conclusions** The pronounced difference of flexion instability between PLC retaining and sacrificing cannot be eliminated only by a different tibial slope, but it is necessary to modify the femoral design with a posterior condylar offset increase. We analysed several solutions to this inconvenient, but we believe that the surgeon should have the opportunity of selecting femoral components, in PS-TKA, with standard size and with posterior condylar offset 2 mm thicker.

**Euthanasia of a painful prosthesis**

F. Centofanti[1], L. Fisichella[3], M. Ciotti[1], R. Orani[1], P. Ditto[3], A. Cambria[3], M.A. Rosa*[3]

[1]Istituto Codivilla-Putti Cortina D'Ampezzo (Cortina D'Ampezzo, IT);
[2]Università degli Studi di Messina (Messina, IT);
[3]Scuola di Specializzazione in Ortopedia e Traumatologia, Sezione di Ortopedia e Traumatologia, Dipartimento Specialità Chirurgiche, Università degli Studi di Messina (Messina, IT)

**Introduction** The "painful" prosthesis is one of the biggest problems in orthopaedic surgery. Opinions about possible diagnostic-therapeutical strategies are contrasting. The aim of this work is to evaluate if all the "painful" prostheses have to be considered as infected.
**Materials and methods** 257 cases of "painful" prostheses were studied from January 1999 to June 2011; taken out from a series of 1419, the average age of patients was 69 years old. Patients did not show any radiographic sign of infection, they had no relevant increase in haematic indexes of phlogosis, the cultures were all negative. Tc99 and marked leukocytes scintigraphies were doubtful. The most relevant element, which is showed in the Index Knee Score, was the worsening of pain. With the traditional methods of instrumental diagnostics and laboratory, the execution of ELISA test on 110

patients is in progress. Every patient has undergone cycles of antibiotic therapy (90 % responsible germ *Staphilococcus aureus*) in local and parenteral administration.

**Results** 88 % (226) of these cases involved the knee, while 12 % (31) the hip. The ELISA test allows us to identify precociously the prosthetic Staphylococcus Aureus infection and to evaluate the efficiency of the antibiotic treatment, through the search for Igm pointed towards slime antigens. Six infiltrative cycles of local antibiotics were performed in an 18 months' period, as final treatment in 75 % of cases (192). In 25 % of cases (n = 64) the infiltrative therapy can be considerated propaedeutic for a surgery of two-stages re-implantation, using an antibiotic loaded prosthesis-spacer prepared directly by the surgeon in the operating theatre. A gram + germ (*Staphylococcus Aureus/Epidermidis*) was identified in 2/3 of cases; in 1/3 there was no development. After a follow-up between 8 months and 5 years, the infection did not relapse in 89 % of cases (n = 58), while in 11 % (7) there was a renewal of the sore symptomatology. In relapsed ones, the pathogenic germs were not isolated.

**Discussion** In authors' experience the "painful" prostheses were treated with six cycles of an antibiotic therapy, in a parenteral and/or articular administration. The latter one allows the reduction or the disappearance of the painful symptomatology. This therapy is then restarted after two-stage re-implantation, when a painful symptomatology reappears and it is used as a periodical, cyclical and protracted therapy. The ELISA test (positive in the 90 % of 110 analyzed cases) seems to allow a precocious individualization of the Staphylococcus Aureus as the main germ responsible for infection.

**Conclusions** Not any infected prosthesis is painful and according to author's opinion, all painful prostheses are recognized as infected.

### *AT02—BIOTECHNOLOGIES*

### Arthroscopic meniscal scaffold implantation: early clinical results at 20-month follow-up

R. Giovarruscio*, V. Condello, V. Madonna, F. Cortese, C. Zorzi

Sacro Cuore Don Calabria (Negrar, Verona, IT)

**Introduction** The treatment strategies for meniscus pathology have been evolving from resection to preservation. Removal of all or part of the meniscus leads to degenerative changes of the articular cartilage and subsequent clinical symptoms. For this reason some authors evaluated the function of meniscal regeneration through a tridimensional synthetic scaffold. The goal of this study was to evaluate the ability of a new scaffold (Actifit Orteq London, UK) to reduce pain, to improve function and quality of life.

**Materials and methods** Since 2010 we have treated 28 patients with ActifitR meniscal scaffold with average age of 40 years and 8 months (16 to 55 years). The mean follow-up was 11 months (6 to 20 months). There were 26 m and 2 f; 4 patients received a lateral meniscus implant and 24 a medial one. The implant of ActifitR was associated with a valgus osteotomy (HTO) in 4 cases and with supracondylar femoral varus osteotomy (DFO) in 1 case, in both with Puddu's plate. In 4 patients was performed reconstruction of the anterior cruciate ligament (ACL) with hamstrings. Non-weightbearing was recommended for the first 3 weeks. Partial weight bearing was permitted from week 4 onwards.

**Results** Following meniscal scaffold implantation, good–excellent results are achieved in nearly 80 % of cases. The IKDC showed 10 patients in group A, 13 in group B and 5 in group C. At the Lysholm score 10 had excellent results. All patients highlighted a measurable decrease of pain, an improvement of function and quality of life.

**Discussion** Implantation of meniscal scaffold combined with ACL reconstruction or correction of the limb malalignment achieved better results. Worse results are associated with degenerative changes of the involved compartment (III or IV0) or with uncorrected axial deviation (3-7 grade).

**Conclusions** Despite a short follow-up, implantation of ActifitR meniscal scaffold represents a reasonable treatment for symptomatic partial meniscectomized knee with early degeneration of articular surface. This device in a short term follow-up can decrease pain, increase knee function, allows pain free activities of daily living.

### Antibacterial activity of platelet gel: analysis, synergy with antibiotic, employment in orthopaedics surgery

J.M. Taglioretti*[1], B. Osnaghi[2]

[1]C.T.O.-I.C.P. (Milan, IT);
[2]Ospedale di Magenta (Magenta, IT)

**Introduction** Sepsis prevention in orthopaedics is a challenge which has got harder and harder. Contradictory scientific evidences on platelet gel (PLG) role against infections appeared. An in vitro study aiming at estimating the antibacterial efficiency of PLG obtained from whole blood of 20 donors versus neaninful bacterial stocks of *Staph. a.* and *Esch. c.* and synergy with chosen antibiotics was carried out in cooperation with the Department of Orthopaedics, Transfusion Service, Microbiology Service at Magenta Hospital (Milan).

**Materials and methods** 100 µl of suspension culture of *Staph. a.* ATCC 25923 and of *Esch. c.* ATCC 25922,0,5 McFarland were inoculated with given amount on antibiotic (Vancomicine-Cefoxitine-Gentamicine) to estimate the concentration inhibiting 30–50 % of bacterial charge. Then the culture were inoculated for each bacterial species: (1) with the selected antibiotic only, (2) with PLG, (3) with antibiotic + PLG.

**Results** The study put in evidence antibacterial activity of PLG thus proving its dependence on time and concentration when starting. In a culture of 20,000,000 UFC/ML of growing *Staph. a.* the addition of 200 µl of PLG has got bacterial-statical action for 2 h 40', increasing to 4H30' by adding 10 µl of Cefotixine, and to 5 h 45' with Gentamicine or Vancomicine. In a culture of 1,000,000. UFC/ML of growing *Esch. c.*, addition of 20 µl of PLG has got bacterial-statical action for 1H20', greater than Gentamicine and than association.

**Discussion** The bacterio statical action of PLG would become evident in corresponding times of critical phase of bacterial adesivity thus preventing its irreversibility which to the formation of Biofilm.

**Conclusions** The employment of PLG during the prosthetic surgery makes the implant safe both because it quickens its osteointegration and because it prevents infections.

### *AT03—HIP*

### The stability femoroacetabular impingement: definition and treatment

V. Bellotti*, E. Astarita, F. De Meo, C. Cardenas, M. Ribas

Instituto Universitario Dexeus (Barcellona, SP)

**Introduction** The stability femoroacetabular impingement (FAI) is a type of hip impingement of less frequent observation. We describe aetiology, diagnosis and treatment based on many years experience of a national center of reference in treatment of hip diseases.

**Materials and methods** A retrospective study over 100 cases arthroscopically, mini open and surgical dislocation operated during the last 8 years.

**Results** We describe middle term follow-up treatment results and success rate depending on preoperative articular degeneration.

**Discussion** The stability FAI is a conflict model in which centrifugal forces act on chondral and capsulo-labral structure at acetabular level, in a contest of hip residual dysplasia. In case of severe dysplasia it would be useful an aetiological treatment with periacetabular osteotomy associated with a contemporary or deferred labral reconstruction.

**Conclusions** In this series of over 100 cases of stability FAI we observed that it is possible to obtain satisfactory results by surgical treatment. The results depend on the type of treatment that best fit with the preoperative lesion severity.

### Ultrasound-guided hyaluronic acid injection in symptomatic treatment of hip osteoarthritis: "Ortobrix" prospective cohort study

G. Logroscino*[1], E. Pagano[1], A. Ziranu[1], V. Ciriello[1], A. Bella[2], M. Bisignani[3], M. Calderaro[4], D. De Amicis[5] F. Mariottini[6], O. Moreschini[7], A. Migliore[8]

[1]Dipartimento di Scienze Ortopediche e Traumatologiche, Università Cattolica-Policlinico A. Gemelli (Rome, IT);
[2]Istituto Superiore di Sanità (Rome, IT);
[3]I Divisione di Ortopedia, Ospedale CTO (Rome, IT);
[4]Unità Operativa di Ortopedia, Ospedale S. Pietro Fatebenefratelli (Rome, IT);
[5]Unità Operativa di Ortopedia, Ospedale S. Salvatore (Pesaro, IT);
[6]Policlinico Casilino (Rome, IT);
[7]Dipartimento di Scienze dell'Apparato Locomotore, Università La Sapienza (Rome, IT);
[8]Unità Operativa di Reumatologia, Ospedale S. Pietro Fatebenefratelli (Rome, IT)

**Introduction** Intraarticular hyaluronic acid injections have been advocated for the treatment of symptomatic hip osteoarthritis. To evaluate the effectiveness of ultrasound-guided hyaluronic acid injections in the treatment of coxalgia/hip osteoarthritic patients.

**Materials and methods** 176 hip osteoarthritic patients were treated with intraarticular ultrasound-guided hyaluronic acid injections (Fidia-Hyalubrix) for 48 months. Patients were evaluated by six independent orthopaedic surgeons. Clinical data (age, BMI, VAS, Lequesne index, clinical history and objectives, use of NSAIDs, and X-rays) were collected. Each surgeon decided whether surgery was indicated. Patients for whom has been decided a surgical indication from at least four of the six surgeons were reassessed at 48 months, in order to evaluate the effectiveness of the conservative treatment. Statistical analysis was performed.

**Results** At 48 months, only 18 % of the population underwent hip surgery. Of those who were considered for prosthetic treatment by at least four out of six orthopaedic surgeons (n = 93) only 33.5 % underwent surgical treatment. The conservative treatment has shown a global efficacy of 66.5 %.

**Discussion** The results showed significant efficacy of hyaluronic acid injections treatment in delaying surgery. The technique allows the ultrasound-guided intraarticular injection of the drug with extreme precision and safety. The reduction of pain may justify the results observed at four years. While presenting some limitations (not prospective, not-comparative study) this is one of the first studies assessing the efficacy of hyaluronic acid hip injection in a significant cohort of patients.

**Conclusions** The hyaluronic viscosupplementation with ultrasound-guided technique is effective in the conservative treatment of hip osteoarthritis. The reduction of symptoms can delay the surgery and may be a viable method of treatment in younger patients, those in whom surgical treatment appears contraindicated, or of those for which is acceptable to postpone surgery. Fast learning-curve and low costs are additional advantages of this technique.

### The influence of the neck resection level and the prosthetic femoral head diameter on the range of motion: a computer simulation

P. Dalla Pria[1], S. Funaioli*[1], M. Nicolai[2], F. Ravasi[3], P. Esopi[4]

[1]SAMO SpA (Bologna, IT);
[2]Università degli Studi di Bologna (Bologna, IT);
[3]AO Ospedale di Circolo (Melegnano, Milan, IT);
[4]ULSS 13 Mirano, Ospedale di Dolo (Dolo, Venice, IT)

**Introduction** So far, in neck preservation hip prostheses the resection level has scarcely been correlated to the resulting Range of Motion (ROM) and there is no general consensus about the optimized level. On the contrary, it is commonly believed that bigger the head, larger the ROM. This is due to the fact there is a clinical evidence of decreasing of the dislocation rate when large heads are used. However, it must be considered that dislocation is a result of two concurrent mechanical actions: the ROM and the jumping distance. The aim of this study is to evaluate the influence of the head diameter and of the neck resection level in the restored ROM.

**Materials and methods** Sawbones Femur and Pelvis were CT scanned. The Dicom files were then converted to a Computer Aided Design (CAD) file in order to manage the graphical models and simulate the joint kinematics. Four models were considered: (1) anatomical Joint (no prosthesis); (2) standard stem (total neck resection); (3) short stem with total neck preservation; (4) short stem with partial (50 %) neck preservation. For each model maximum joint angles were measured (flexion–extension, abduction–adduction, internal-external rotation and high dislocation risk daily living activities). Femoral head diameters of 28, 32, 36, 40 mm were used. The values of the anatomical Joint were compared to the Literature data to validate the CAD model.

**Results** The anatomical Joint exhibited the following results: Flex/Ext = 120°/69°; Abd/Add = 50°/48°; Int/Ext = 91°/54°. The widest angular range was obtained with model 2: Flex/Ext = 131°/69°; Abd/Add = 66°/50°; Int/Ext = 122°/71°. Model 4 ranked slightly after model 2: Flex/Ext = 122°/76°; Abd/Add = 66°/49°; Int/Ext = 119°/61°. Model 3 exhibited the narrowest ROM: Flex/Ext = 107°/33°; Abd/Add = 49°/35°; Int/Ext = 90°/32°. The same trend is evidenced simulating the critical daily living activities. The above data were obtained with 36 mm head. However, they are almost independent from the diameter.

**Discussion** Total neck resection and partial neck preservation enhance theoretical ROM respect to the physiological model. On the contrary, total neck preservation can reduce the ROM. Bigger head is not really proving any help since obtained values are independent from the diameter.

**Conclusions** It is possible to conclude that femoral head diameter does not influence ROM. The decrease of dislocation risk with large heads is directly related to a longer jumping distance. According to the simulation the total neck preservation reduces the theoretical ROM.

**Retentive cup: an experience of 456 cases in "great ancient" people**

P. Ghiggio*, U. Scarlato, R. Serracchioli, A. Boschetti, A. Devecchi

ASLTO4 (Ivrea, IT)

**Introduction** The peculiarity of a retentive acetabular component is to be provided of a plastic ring producing an antiluxation effect of the femoral prosthesis component. The ring allows the transfer of forces to bone and acetabular interface. Capsular laxity, muscles' strengthness and neurological diseases are an important cause of unsuccess in "great ancient" people.

**Materials and methods** In 2003 we chose this type of first implant in a series of patients with medial femoral neck fracture. After a period of training of this implant in neurological patients with frequent components luxation, we extended the use to older an Alzheimer disease suffering patients. Following this way we had no more dislocation. In our casistic we have evaluated proximal femural fractures. The mid-age was 67.5 years old (34 to 101). Females represented 4.1. The "great ancient" with medial fractures considered for this work were 701. Among these, 556 were operated with a total hip implant. The other ones did not perform a surgical procedure for general conditions or underwent to an osteosynthesis. The retentive implant was used in 456 cases. These ones had a fracture: AO 31 B1 in 26 cases, Ao 31 B2: 115 cases; AO 31B3 315 cases. The couple was performed with a cemented self-locking stem in 350 cases. In 106 we recurred to a Hidroxyapatite-coated and squared stem.

**Results** Our aim was to evaluate mechanical complication only (dislocations and loosening). We had four cases of luxation: two in an implant with a 22 mm diameter head, no more used. And two with a 28 mm head, probably due to an intraoperative mistake. One case had a stem loosening (cementation mistake?).

**Discussion** This type of retentive implant is not too much expensive than a biarticular one. The reduced risk of dislocation offsets the cost of the implant because it does not occur more days in Hospital to resolve this eventual complication. The surgical timing and blood loosening are quite similar as well.

**Conclusions** A total hip implant with a minimal mechanical risk consents a best postoperative nursing with an early mobilization time for the patient either in sitting or in vertical position. These are the goals for a successful hip surgery in very ancient people.

### *AT04—ACUTE CAPSULO-LIGAMENTOUS INJURIES*

**Drilling the femoral tunnel during ACL reconstruction: trans-tibial versus antero-medial portal techniques: a cadaveric study**

C. Tudisco*, S. Bisicchia

Università di Roma Tor Vergata (Rome, IT)

**Introduction** A wrong position of bone tunnels, in particular on the femoral side, is one of the most frequent causes of a failed anterior cruciate ligament (ACL) reconstruction. Several studies demonstrated that drilling the femoral tunnel through the antero-medial portal (AMP) allows a more anatomical placement on the lateral femoral condyle and higher knee stability than trans-tibial (TT) reconstruction.

**Materials and methods** In 12 cadaveric knees the femoral tunnel was drilled both with the TT and the AMP techniques. With appropriate landmarks inserted into bone tunnels, X-rays were taken in AP and lateral views and the direction and length of the tunnels were determined. Subsequently, in 6 knees the ACL was reconstructed with the TT technique and in 6 knees with the AMP technique with an autologous STG graft. Knee stability was evaluated with KT-1000 arthrometer and pivot shift test, comparing for both techniques the difference between pre- and post-operative values. Finally, all the knees were dissected to enhance better vision of the insertion of the reconstructed ligament.

**Results** AMP technique led to a better placement of the femoral tunnel both in coronal and in sagittal plane. The mean angles between tunnels was 25 degrees and 65 degrees in coronal and sagittal planes respectively ($p < 0.05$), with improved stability at pivot shift, but not at KT-1000.

**Discussion** The anatomical and clinical results reported in literature about TT and AMP techniques are controversial, but most of the studies reported better results for AMP technique, especially for rotational stability.

**Conclusions** Our cadaveric study showed that the AMP technique provided better tunnel placement on the lateral femoral condyle, both in coronal and sagittal planes with an improvement in the rotational stability of the knee.

**Long-term clinical and radiographic outcomes of anterior cruciate ligament reconstruction with BPTB: a prospective study**

F. Dettoni*[1], R. Rossi[1], F. Castoldi[1], D. D'elicio[1], M. Bruzzone[1], D. Blonna[1], D. Bonasia[2], M. La Russa[1], A. Marmotti[1]

[1]Ospedale Mauriziano (Turin, IT);
[2]Ospedale CTO (Turin, IT)

**Introduction** The aim of this prospective study was to evaluate 57 patients who underwent an ACL reconstruction using the patellar tendon (BPTB) at a long-term follow-up (7.9 years, range 4.2–10.7), assessing objective and subjective treatment satisfaction, radiographic appearance and the degree of arthritis of the knee.

**Materials and methods** All patients were evaluated using: (1) the International Knee Documentation Comitee (IKDC) Score and the Lysholm Score for objective evaluation; (2) the Visual Analogic Scale (VAS), the Tegner Score, the Knee Injury and Osteoarthritis Outcome Score (KOOS) and Short-Form 36 (SF-36) for patients' subjective evaluation; (3) a clinical evaluation, including the most commonly used clinical tests: Lachman, anterior and posterior drawer, varus and valgus stress, and pivot shift (jerk) tests; (4) the amount of anterior instability, measured with the KT-1000 device; (5) the amount of knee arthritis, on a plain X-rays, according to Fairbanks' classification.

**Results** The mean scores were: VAS: 2.02. Tegner: 5.51. KOOS: symptoms 22.84; pain 32.47; activities of daily living 65.96; sport 16.35; quality of life 12.37. SF-36: physical functioning 92.46; physical role functioning 85.09; pain 77.75; general health 73.11; Vitality 68.86; social role functioning 80.96; emotional role functioning 84.12; mental health 77.75. Lachman +/++ : 34.48 %. CAD +/++ : 10.34 %. v–v stress +: 7.01 %. pivot shift +/++ : 61.4 %. Lysholm: 90.26. KT1000: 0.78 mm. Fairbanks: moderate to severe arthritis: 78.94 %.

**Discussion** We collected satisfactory outcomes at 8 years follow-up after an ACL reconstruction using BPTB. The most interesting findings raise from the comparison between subjective and objective evaluation. Most of the patients returned to pre-injury sports activity. The amount of pain and patients' satisfaction were good to excellent. While patients were highly satisfied by their knees' function, we observed a residual rotatory instability that was not noted by the patients. KT1000 measures confirm that ACL reconstruction reduces the anterior tibial sag and instability, but does not restore the normal knee function. WE observed radiographic signs of arthritis in most of the patients, but this was not associated with pain nor functional limitations.
**Conclusions** ACL reconstruction using BPTB is a treatment of proven efficacy. Our findings demonstrate that the treatment is associated to a good patients' satisfaction with limited pain at 8-year follow-up. A residual rotatory instability was detected, but it was not noted by the patients. We also found a high incidence of arthritis, though it is not associated to pain nor any functional limitation.

### New classification of soft tissue constraint lesions in complex elbow instability

G. Giannicola*, D. Polimanti, F. Sacchetti, M. Scacchi, F. Postacchini

"Sapienza" Università di Roma (Rome, IT)

**Introduction** Soft tissue constraint lesions (STCL) are a consistent finding in complex elbow instability (CEI) and they have to be taken into account during diagnostic evaluation and surgical treatment. However, the prevalence and pathoanatomy of acute STCL of the elbow are not well documented in literature. The aim of this study was to analyse prospectively the prevalence and pathoanatomy of STCL in CEI and to provide a comprehensive classification system of these injuries.
**Materials and methods** In 47 patients with CEI, lateral compartment injuries were identified by direct visualization, while medial compartment lesions were diagnosed using fluoroscopy when not surgically addressed. Ligament injuries were classified into two groups: simple and complex lesions. Simple lesions: TypeP or proximal avulsion, TypeM or mid-substance lesion and TypeD or distal avulsion. Complex lesions: TypePM (TypeP + TypeM), TypeDM (TypeD + TypeM), TypePMD (TypeP + TypeM + TypeD). Postero-lateral capsule injuries (PLCI) were defined as small if consisting in a detachment $< 1$ cm from the postero-lateral aspect of the distal humerus, and as large when $> 1$ cm. Proximal/distal ligament avulsions and PLCI were further classified as "a" when associated with a bone fragment avulsion $> 5$ mm. Common extensor origin (CEO) and flexor pronator origin injuries were diagnosed in the presence of a lack of continuity greater than 50 % of the muscle–tendon component. Reliability of classification was assessed in 25 cases by 3 surgeons who were asked to classify each soft tissue constraint injuries on intraoperatively images. The K coefficient was calculated.
**Results** Ligament lesions were found in 96 % of patients. The lateral collateral ligament showed a simple type P, M, and D-a in 19, 13 and 2 % of patients, respectively. Two patients showed a type P-a lesion. Complex types PM, DM, PDM were found in 47, 6 and 4 % of cases, respectively. Small and large PLCI were found in 49 and 17 % of patients, respectively. A medial collateral ligament injury was present in 45 % of patients. CEO injuries were present in 21 % of patients. The reliability was good ($K = 0.772$).
**Discussion** The high prevalence of observed STCL suggests a careful evaluation of these injuries during the diagnostic and therapeutic algorithm of CEI. A comprehensive and reproducible classification may be useful to guide the surgeon in the reconstruction of STCL and to compare the results of different surgical techniques.
**Conclusions** This study has proposed a new comprehensive and reproducible classification of STCL in CEI.

### ORAL COMMUNICATIONS

### *C19—HIP 3*

### Evaluation of the effects of the stem design on periprosthetic bone remodelling in total hip arthroplasties

P.D. Parchi*[1], G. Ciapini[1], J. Castellini[1], A. Vigorito[1], S. Marchetti[1], S. Maffei[2], M. Lisanti[1]

[1]I Clinica Ortopedica Universitaria (Pisa, IT);
[2]Istituto Fisiologia Clinica, CNR (Pisa, IT)

**Introduction** Total hip arthroplasty is one of the most performed procedures in orthopaedic surgery. Implantation of a prosthesis determines changes in the distribution of loads on the host bone, and this phenomenon, known as stress-shielding is related to the biomechanical characteristics of the implant and it involves the proximal portion of the femur by reducing the mechanical strength and ability to withstand the transmitted loads. The aim of our study is to demonstrate how the use of a short hip stem reduces the stress-shielding phenomenon to the proximal femur compared to stems with a diaphyseal fixation.
**Materials and methods** The study analyzed 40 patients undergoing hip prosthesis surgery at the Ist Orthopedic Division of Pisa University between December 2008 and January 2010. The patients were divided into two groups according to the type of femoral stem: 20 with a short stem with a metaphyseal fixation (Metha B-Braun) and 20 with straight stem with a diaphyseal fixation (Hipstar Stryker or Oak Permedica). Each patient was subjected to analysis of periprosthetic bone mineral density by a Bone Densitometry (DEXA: Dual-emission X-ray absorptiometry with the metal removal software) at 0, 6, 12, 18 and 24 months, following a protocol based on the evaluation of the changes of bone density in the 7 Gruen zones.
**Results** The results obtained do not show significant differences in the curves of periprosthetic bone resorption at the level of the greater trochanter (Zone 1) at the apex of the stem (Zone 4), of the lateral regions (Zones 2–3) and medial regions of the (Zones 5–6). A different trend in the variation of periprosthetic bone mineral density occurred in zone 7 (Calcar) with a better result for the group with stem Metha [post-op. 1.08 g/cm$^2$-2y 0.914 g/cm$^2$] compared to the group with straight stem [post-op. 1.015 g/cm$^2$-2y 0.774 g/cm$^2$].
**Discussion** The data obtained from our study are in agreement with other studies in the literature which demonstrate how the use of short stems allow to preserve during the time the metaphyseal bone stock at the level of the calcar, reducing the stress-shielding phenomenon at the proximal femur.
**Conclusions** Given that the presence of a higher quality periprosthetic bone is associated with a greater implant survival, with less risk of aseptic loosening, we can conclude that short stems can be considered a more durable and a more suitable option especially for young patients.

### The percutaneous compression plate in the treatment of pertrochanteric hip fractures

R. Matteotti*, E. Grosso, R. Sisto, L. Rollero, M. Tarello, S. Vanni, B. Battiston

Traumatologia, CTO Torino (Turin, IT)

**Introduction** As the world population ages, the prevalence of osteoporosis and the incidence of hip fractures will increase dramatically, being responsible for an increase of the health expenditure. Hip fracture surgery is associated with high post-operative mortality and poor functional results: the excess mortality is 20 % in the first year; of those patients who survive, only 50 % recover their previous ability to walk. Selection of a correct type of implant for fracture fixation is a very interesting challenge. Surgical reduction is currently the preferred treatment. A minimally invasive fixation device such as the Gotfried's Percutaneous Compression Plate may potentially improve patient's outcome.
**Materials and methods** We considered 545 patients with pertrochanteric fracture (AO type 31 A1 and 31 A2) treated in our Department with PCCP between May 2003 and May 2010. Mean age: 80.7. Female: 73 %. We collected the following data: pre-injury and post-operative general health condition with the Parker's mobility score HB pre- and post-operative, number of transfused blood units, mortality, surgical and clinical complications.
**Results** Mortality: 20 % (31 % in males, 16 % in females). The mortality has been bigger in the patients with more than 80 years and in patients with a pre-injury low functional status. Lower Functional status: 65 %, Mean number of transfused blood units: 0.85. Complications were: 4 DVT, 3 infections, 3 surgical failures, 12 cut out, 4 screw loosening.
**Discussion** Fixation of pertrochanteric fractures may be satisfactorily accomplished with either a sliding compression screw plate or an intramedullary nail. The choice depends more upon surgeon preference that on any real difference in functional outcome In patients whose general condition is frequently compromised by severe concomitant medical or surgical conditions, primary objectives in proximal femoral fractures are that there should be minimal operative trauma and blood loss and that walking ability should be, if possible, maintained.
**Conclusions** Our results agree with data in literature as to the clinical outcomes of pertrochanteric fractures. Thus, the PCCP can be considered a good option in the surgical treatment.

### Hip arthroplasty with AMIS technique: learning curve

F. Rivera*, A. Bardelli, F. Leonardi

Dipartimento Ortopedia e Traumatologia, Ospedale SS Annunziata, ASN CN1 (Savigliano, Cuneo, IT)

**Introduction** Anterior approach has a real advantage of inter-muscular and inter-nervous approach. On the contrary, the disadvantage for the introduction of a less invasive technique in the surgery could have a risk of complications due to the introduction of a new technique. Aim of our study is the retrospective analysis of the first 103 hip arthroplasties with anterior approach using AMIS leg holder.
**Materials and methods** Between March 2010 and March 2011, 113 hip arthroplasties has been done in our hospital using AMIS technique. All patients were recorded pre-operatively with clinical evaluation (Harris hip score and Vas) and post-operatively with clinical evaluation (Harris hip scores and VAS with follow-up 1, 3, 6 months, 1 year) and radiographic analysis (follow-up 1, 3, 6 months, 1 year). Preoperative diagnosis was hip arthritis in 96 cases, osteonecrosis of the femoral head in 7 cases. The group of patients included 55 females and 11 males, with the mean age of 66 (range 46–74). The retrospective study includes the analysis of surgical times, the incidence of complications and clinical results regarding single surgeon's learning curve with this approach.
**Results** We have 101 patients with 1 year follow-up. No statistically significant difference was found in surgical times during first 50 cases (79 min) *vs.* other 51 cases (76 min) ($p > 0.5$). We have noticed 2 subsidence cases (2 %) after 3 months without consequences on implant stability, 2 intra-operative neck fissure cases followed by a preventive intra-operative cerclage and 1 intra-operative proximal femur epiphysis fracture case with revision of implant after two days. No statistical significance was found in chronologic distribution of compliance study group.
**Conclusions** In opening phase with AMIS technique, we did not have serious complications, as we can see on literature. Learning curve does not seem longer than other approaches. The use of AMIS leg positioner, which must not be used with an excessive traction but strictly for femur neck cut, is certainly useful for decreasing people in operating room for surgery and to facilitate femur exposition in reproducible form.

### *C20—PAINFUL PROSTHESES 1*

### Management of post-operative pain and bleeding in knee replacements

G.L. Canata*, V. Casale, A. Chiey

Centro Traumatologia Sport Koelliker (Turin, IT)

**Introduction** In total knee replacement surgery, procedures to reduce postoperative pain and bleeding are performed. The objective of this prospective randomized study was to evaluate if, infiltrating tissues with a mixture of Ropivacaine, Clonidine, Ketorolac and Norepinephrine, gave better results than with Tranexamic acid or not infiltrating at all.
**Materials and methods** 96 patients, mean age 70 years (range 47–83), were randomized in 3 groups: each one of which was composed of 32 patients, operated between 2008 and 2009 for total knee replacements. In Group A no infiltration was performed, 11 males and 21 females. In Group B an infiltration of Tranexamic acid was performed in 11 males and 21 females. In Group C, a mixture of the 4 aforementioned drugs was infiltrated, 12 males ad 20 females. Both cemented and non-cemented knee replacements were used; in all cases postoperative drainage was inserted, open 3 h after the end of surgery. A pre- and postoperative evaluation was performed using the Knee Injury and Osteoarthritis Outcome Score (KOOS) with a mean follow-up of 23 months; the difference in the preoperative haemoglobin value and that within the first postoperative week was calculated as a parameter of blood loss. Statistical analysis was performed by T test.
**Results** In Group A the mean postoperative score was 78 (St Dev 19); the mean reduction of haemoglobin compared with the preoperative one was 3,1 g/dl within the first week and blood transfusions were performed in 2 cases. In Group B the mean KOOS score was 83 (St Dev 16), the haemoglobin was reduced to 3.6 g/dl and a transfusion was necessary in 1 case. In Group C the mean KOOS score was 90 (St Dev 15), the haemoglobin was reduced to 3.7 g/dl and 2 transfusions were performed. The differences between the three groups were not statistically significant.
**Discussion** The blood loss in the 3 groups did not result significantly different. The opening of the drainage 3 h after surgery could be a favorable factor. Instead, differences in postoperative pain were observed, less in Group C.
**Conclusions** This study confirms that many factors act in postoperative pain and bleeding. Infiltrating the surgical wound with antalgic and vasoconstrictive substances obtains better postoperative functional results although without statistical significance. There are no differences in blood loss.

### Knee replacement in osteoporotic patients: direct correlation between pain and periprosthetic bone resorption

E. Gasbarra*, P. Pistillo, M. Arduini, L. Saturnino, A. Scialdoni, F. Perrone, N. Cuozzo, U. Tarantino

Università degli Studi di Roma "Tor Vergata" (Rome, IT)

**Introduction** Periprosthetic bone resorption may be one of the main factors affecting the stability of the implant and its clinical outcome, determining the onset of pain. The purpose of this study is to determine a possible correlation between the bone resorption around tibial component in cemented total knee arthroplasty and the development of postoperative pain.
**Materials and methods** In the study were enrolled 30 consecutive patients with a mean age of 65 years (range 55–75), 18 females and 12 males, underwent surgery between January 2010 and December 2010. Patients were evaluated by preoperative lumbar and femoral DXA scan. Both DXA and radiographic examination of proximal tibia were performed before surgery, in the immediately postoperative period at 3, 6 and 12 months after implantation. Presence of pain, during the postoperative follow-up, was evaluated using VAS scale while functional recovery was measured with Knee Society Score (KSS).
**Results** Thirteen patients resulted osteoporotic at lumbar and femoral DXA (9 women and 4 men), 7 were osteopenic (5 women and 2 men), 10 with normal BMD (4 women and 6 men). Nine patients had pain with a median value of 5 (range 4–7) in VAS scale at 6 months; among these, 7 patients showed osteopenic values of BMD. The evaluation by KSS showed a reduction of values obtained in these patients (mean 57, range 43–66) compared to those with no pain (mean 91, range 87–95). Radiographic examinations revealed no signs of loosening in any case.
**Discussion** In our study, 77.7 % of patients who referred pain during the clinical evaluation showed an osteoporotic BMD. The evaluation with KSS showed the best average results in patients without pain and a better functional recover. The comparison of results obtained by DXA and clinical study showed a correlation between reduced BMD and the occurrence of postoperative pain, without visible changes on radiography. The results obtained suggest that a reduced bone quality may cause a prosthetic component micromotion resulting in a reduced stability generating pain.
**Conclusions** The DXA method is therefore a useful tool to identify patients with an higher risk of loosening of the implant, showing periprosthetic bone changes not yet visible on X-rays. Using this method it is possible to administrate an osteotropic therapy that promotes implant osseointegration, avoiding a possible mobilization.

### Retrospective study on about 208 systems of knee prosthesis: the role of the pseudo-patella baja in pain and ROM reduction

S. Giannotti*, V. Bottai, G. Dell'Osso, L. Palmeri, N. Cazzella, G. Bugelli, G. Guido

Clinica Ortopedica, Università di Pisa (Pisa, IT)

**Introduction** One of the possible complications of total knee arthroplasty (TKA) is a patella baja. We must distinguish between true patella baja (PB), when the patellar tendon is short, and the pseudo-patella baja (PPB), when the joint line rises up. The post operative PB could have different etiologies, already treated in the literature; the PPB may be the result of an excessive resection of tibial or femoral component. These eventualities require the placement of a polyethylene component of greater thickness. The purpose of this retrospective study is to analyze the clinical and radiographic results obtained in patients with TKA, searching for PPB and its correlation with the painful prosthesis.
**Materials and methods** We reviewed 158 patients, for a total of 208 implants who underwent primary TKA in our Clinic since 1998 to 2011. The mean follow-up was 39.6 months (min. 4, max. 163), 49 men, 109 women, average age of 72.6 years (min. 36, max. 87). In our study we used the following scores: (1) Insall-Salvati (2) Modified Insall-Salvati (3) Blackburne-Peel, and (4) Caton-Deschamps; in assessing the actual presence of a PPB, however, we observed only the last two tests, the only ones that analyze the distance between the patella and new tibial plateau. We also rated the pain reported by patients using the Knee Society Score (KSS) and the WOMAC score.
**Results** About 208 implants, 68.5 % didn't show elevation of the joint or shortening of the patellar tendon (group A). In 32.5 % of the patients was observed the presence of a PPB (group B). We observed that KSS and the Range of Movement (ROM) were lower in group B compared to group A. Furthermore, patients in group B showed significantly more severe pain than patients in group A.
**Discussion** The evaluation of pseudopatella baja is an index of clinical-functional lower score and accentuated pain, in addition to being an index of variation of the height of joint line obtained surgically in the implant. That is because PPB reduces the ROM and the lever arm, increases energetic cost and promotes the impingment of patella against the tibial polyethylene.
**Conclusions** Careful preoperative planning and the help of pre-navigation can reduce the risk of PPB, with significant clinical and functional benefits.

### *C21—SHOULDER AND ELBOW 5*

### Lesions of the rotator cuff. Arthroscopic versus open mini: our experience

R. Lupo*, S. Rapisarda, G. Palmisciano, R. Arcidiacono

ASP Agrigento, U.O. di Ortopedia e Traumatologia, Ospedale S. Giovanni di Dio (Agrigento, IT)

**Introduction** The purpose of this work is to analyze the clinical results obtained from the use of surgical techniques of arthroscopic assisted mini-open and total arthroscopic surgery in a homogenous group of patients for the repair of small and medium-sized lesions of the rotator cuff.
**Materials and methods** We have treated from November 2002 till June 2011, 392 patients, 155 women–237 men, with a follow up on average of 4 years, 189 underwent surgery with arthroscopic assisted mini-open technique (73 women, average age 50.9, 116 men, average age 54.2), while in 203 patients, the technique has been entirely arthroscopic (85 women, average age 50.5, 118 men, average age 55.3). The clinical evaluation was based on the Simple Shoulder test, the Constant score, associated with a preoperative study of X-rays in specific projections and on a RMN. In 60 % of the cases, a subacromial decompression with acromioplasty was used, in 50 % a tenotomy-tenodesis of the biceps, in 20 % a Mumford. In the mini-open techniques the use of the arthroscope allowed us to perform in necessary cases acromioplasty and Mumford without disconnection of the deltoid.

**Results** In terms of the Constant score there was not a significant difference between the two groups respectively arthroscopy (average 85) mini-open (average 82). The period for a proper rehabilitation were comparable, whilst it was noticed there was a better tolerance and reduction of pain both in the immediate postoperative period and during the process of rehabilitation in patients operated with fully arthroscopic technique.
**Discussion** The purpose of these studies aim to underline the difference between the two groups in terms of clinical results, of post operative pain and the rate of ri-rupture. In our studies we have not noticed significant differences between the open technique and the arthroscopic technique in terms of clinical outcome. The only significant differences highlighted were in a reduction in pain during the first postoperative week, and a slower progression of fatty muscle degeneration in patients treated with arthroscopy.
**Conclusions** In conclusion, having found no significant differences between the two groups, arthroscopic and mini-open, we can say that both procedures are effective in the surgical treatment of rotator cuff lesions as regards the final result. On the other hand it should be emphasized that the arthroscopic technique is preferable for better appearance of the surgical incision, less postoperative pain and a better rehabilitation, and especially because the same technique allows the treatment of associated injuries.

### Cuff injury treated with patch: morphological and functional analysis

S. Giannotti*[1], M. Ghilardi[1], G. Dell'osso[1], L. Magistrelli[1], G. Bugelli[1], F. Di Rollo[1], G. Ricci[2], R. Calabrese[2], G. Siciliano[2], G. Guido[2]

[1]Clinica Ortopedica, Università di Pisa (Pisa, IT);
[2]Dipartimento Neuroscienze, Clinica Neurologica, Università di Pisa (Pisa, IT)

**Introduction** In the pathology of the rotator cuff the possibility to obtain an efficient repair of the tendon injury in many cases is conditioned not only by the type and size but also the quality of the tissue; in fact it is possible to obtain a complete repair but the tendon tissue cannot be optimal for a correct functional recovery. The need to solve the most of these situations has led us to use the scaffolds that have the ability to fill the tendon residue gap but also the possibility of being used as augmentation to reinforce the structure tendon. We therefore wanted to evaluate our experience with the use of the Zimmer Collagen Repair Patch.
**Materials and methods** The cases treated with this scaffold are 18 with a minimum follow-up of 8 months and a maximum of 43 months, they were clinically evaluated preoperatively and postoperatively with ASES and Constant scores. Reassessed at distance with ultrasound, MRI to evaluate the change in muscle trophism (Goutallier evaluation) and electrophysiological study to evaluate the motor unit recruitment and muscle functional status.
**Results** The clinical results in constant and ASES scores were excellent with an average value above 80 points. The ultrasound evaluation and with MRI shows first of all the restoration of the continuity tendon with the average thickness of 4 mm and also with the imaging there was a reduction of the fatty component with respect to the preoperative control. The results EMG are in progress.
**Discussion** By following patients postoperatively we found that the resolution of pain is rapid and constant, while the recovery of muscle strength occurs within 4–6 months after surgery.
**Conclusions** Following the experience we can say that this collagen patches could represent a very important tool for the surgeon's shoulder in order to resolve situations where the tendon repair alone does not ensure proper recovery of the gleno-humeral joint function.

### Discovery elbow system: short term results in distal humerus fractures and post-traumatic osteoarthritis

G. Giannicola, D. Polimanti*, F. Sacchetti, M. Scacchi, E. Manauzzi, F. Postacchini

"Sapienza" Università di Roma (Rome, IT)

**Introduction** Currently, Coonrad-Morrey device represents the gold standard of linked total elbow prosthesis (TEA). However, scarce information are available in literature about other recently introduced linked TEA. The aim of this study is to evaluate short term results of Discovery Elbow System (DES) in a group of patients with acute fractures of distal humerus or post-traumatic osteoarthritis.
**Materials and methods** There were 8 men and 14 women mean aged 68 (range 50–88) years. 8 patients, mean aged 80 years, had an acute comminuted distal humerus fracture, while 14 patients presented a severe post-traumatic osteoarthritis with chronic pain and stiffness. Post-operative management consisted in active and passive motion started 2 days after surgery and indometacin was administered for 5 weeks in all patients. Clinical outcomes were evaluated with MEPS score. A pre-operative clinical evaluation was performed with MEPS in patients with posttraumatic osteoarthritis.
**Results** The mean clinical and radiographic follow-up was 25 months (range 6–37). One patient was lost at the follow-up. The functional arc of motion at the last follow-up (30–130 in E/F and 50–50 in P/S) was achieved in 18 out of 21 patients. The average extension loss was 19° (range 0–50) and the average flexion was 138° (range 130–145); average forearm rotation was 80° of pronation and 85° of supination (range 80°–90°). The average MEPS score was 93 points (range 65–100). In patients with post-traumatic osteoarthritis, average MEPS was 96 with a postoperative increase of 60 points. In fractures group average MEPS was 92. In all patients, according to MEPI, there were 17 excellent, 3 good, 1 fair results. Complications were two patients with a transitory ulnar nerve neuropathy and one alcoholic patient, with a fair result, with a periprosthetic humeral fracture and infection. Progressive radiolucent lines and implant loosening were not observed. No significant difference was observed between results of patients treated for acute fracture and patient treated for post-traumatic stiffness.
**Discussion** Preliminary clinical and radiographic results of DES are encouraging. Our results show the short-middle term validity of this prosthesis in the treatment of acute fracture and in posttraumatic stiffness. The anatomic design and the advanced instrumentation simplified the surgical technique. Patient selection, correct cementation, accurate surgical technique and careful postoperative management are mandatory to achieve an optimal result. Further studies with longer follow-ups are necessary to confirm the survivorship of this prosthesis.

### *C22—SPINE 4*

### Lyon bracing in adolescent females with idiopathic scoliosis: assessment of results according to the SRS committee on bracing and non-operative management standardization criteria

A.G. Aulisa*, V. Guzzanti, M. Giordano, C. Perisano, G. Scudieri, L. Aulisa

U.O.C. di Ortopedia e Traumatologia, Ospedale Pediatrico Bambino Gesù (Rome, IT); Dipartimento di Ortopedia, Università Cattolica (Rome, IT)

**Introduction** The Lyon brace is commonly prescribed in many European countries and is considered to be an effective means for the conservative treatment of scoliosis. The brace is based on the three-point pressure system and also applies derotational forces to the spine, with the main thoracic plate pushing the hump from the posterior convexity toward the anterior concavity of the scoliotic curve. The purpose of the present study was to evaluate the efficacy of Lyon bracing for the conservative treatment of adolescents with idiopathic thoracic curves. Analyses were performed in a case series selected on the basis of the Scoliosis Research Society (SRS) Committee on Bracing and Non-operative Management Standardization Criteria.
**Materials and methods** Sixty-eight adolescent females (mean age 11.8 ± 0.5 years) with thoracic curve and a pre-treatment Risser score ranging from 0 to 2 were enrolled. All patients were prescribed with full-time Lyon bracing. The minimum duration of follow-up was 24 months (mean: 36.4 ± 27.0 months). Antero-posterior radiographs were used to estimate the curve magnitude (CM) at 5 time points: beginning of treatment (t1), one year after the beginning of treatment (t2), intermediate time between t1 and t4 (t3), end of weaning (t4), 2-year minimum follow-up from t4 (t5). Three outcomes were distinguished: curve correction, curve stabilization and curve progression.
**Results** The CM mean value was 34.1 ± 7.4 SD at t1 and 23.1 ± 10.4 SD at t5. Curve correction was accomplished in 83.8 % of patients, whereas a stabilization curve was obtained in 16.2 % of patients. None of the patients experienced a curve progression.
**Conclusions** The Lyon brace, due to its biomechanical action on vertebral modeling, is highly effective in correcting thoracic curves.

### Stabilization with P.L.F. in surgical treatment of lumbar spondylolisthesis: considerations on a series of 49 cases

A. Vilardi, L. Ciriaco, G. La Rosa, M. Ferlazzo, M.A. Rosa*

Università degli Studi di Messina (Messina, IT); Istituto Clinico Polispecialistico COT (Messina, IT); Istituto Codivilla-Putti (Cortina D'Ampezzo; IT); Scuola di Specializzazione Ortopedia e Traumatologia, Dipartimento Specialità Chirurgiche, Università degli Studi di Messina (Messina, IT); Scuola di Specializzazione in Ortopedia e Traumatologia, Sezione di Ortopedia e Traumatologia, Dipartimento Specialità Chirurgiche, Università degli Studi di Messina (Messina, IT)

**Introduction** The Authors started their analysis by considering the degenerative lumbar vertebral instability determined by the spondylolisthesis. The various methods of surgical treatment of this disease do not direct the orthopaedist towards a standardized technique, although arthrodesis is the main target to reach.
**Materials and methods** From 1996 to 2011 49 patients, aged from 20 to 65 years old, affected with degenerative spondylolisthesis L4-L5 (21 pts.) and L5-S1 (28 pts.) were treated. The surgical treatment, on the posterior approach, was performed with stabilization and arthrodesis with pedicle screws and rods, and autologous graft with morcelized bone; previous laminectomy, if necessary, was executed. For radiographic evaluation authors used the White and Punjabi lumbar instability grid. All patients treated with surgery, were assessed with pre- and post-surgical X-rays, and then annually up to a maximum of 15 years; during the last year 26 patients decided to undergo a new clinical and radiographic control; the result was a clinical follow-up from 1 to 14 years.
**Results** The X-rays control performed on all patients treated with surgery, showed an arthodesis engraftment on 100 % of cases up to a year, with a good maintenance of osteosynthesis also in the later controls (up to 15 years). The clinical analysis performed on 26 patients treated with rods and screws, during the last year, showed a high level of patients (80 %) that were satisfied with better results, with an improvement on the White and Punjabis' score. The remaining cases were either not very satisfied (12 %) or not- satisfied (8 %). 3 % of them were affected with complications during their treatment, after the therapy (postural pain, mobilization of osteosynthesis, discal pathology and very strong mobility of the segments close to arthodesis).
**Discussion** The first treatment of spondylolisthesis is conservative, (psychological evaluation, medical treatment, physiotherapy, corset). The failure of this therapeutic treatment leads to a surgical decision. In the Author's experience, the obtaining of arthrodesis through the provision of autologous morcelized bone, always represents the main goal to reach.
**Conclusions** The authors, after a review of the literature and having studied their own personal cases, conclude that the instrumental technique with bars and screws in lumbar instability allows the quick and safe accomplishment of arthrodesis engraftment, determining a significant improvement in the clinical state of the patients treated with surgery, despite various complications.

### New vertebral fractures after vertebroplasty: risk–benefit assessment

A. Ruggiu*[1], P. Tranquilli Leali[1], M. Spiga[2], F. Muresu[1], C. Meloni[1], A. Zachos[1], C. Doria[2]

[1]Clinica Ortopedica, Università degli Studi di Sassari (Sassari, IT); [2]U.O.C. Ortopedia e Traumatologia, San Martino-Oristano (Oristano, IT)

**Introduction** In the treatment of osteoporotic vertebral compression fractures, vertebroplasty proved to be a safe procedure and effective in a statistically significant reduction in pain symptoms and improving quality of life. To date, the literature has not yet clarified the correlation between this minimally invasive technique and the incidence of new osteoporotic vertebral fractures (VF).
**Materials and methods** In this retrospective study we evaluated the incidence and distribution of new VF in 124 patients, previously treated with vertebroplasty for 193 VF, from 2006 to 2011 at the Orthopaedic Clinic of Sassari.
**Results** The 16.5 % of patients developed new VF at a median time of 8.6 months (range 1–35 months) after surgery. 25 % of these occurred at a level immediately adjacent to the one treated while 75 % at a distance, with the vertebrae near to that treated most frequently involved.
**Discussion** The biomechanical changes resulting from the addition of polymethylmethacrylate (PMMA) into the vertebral body may lead to an increased risk of developing new VF segments adjacent to that treated. This risk may be increased in a statistically significant manner in the presence of an excess of PMMA or a non-uniform distribution in the soma.
**Conclusions** This risk does not appear to be higher than that resulting from the fracture itself. In literature, in fact, has reported an incidence of new VF, for 12 months, 19.2 %, related to the progression of the osteoporotic disease and biomechanical changes caused by the fracture.

### *C23—NEOPLASTIC DISEASES AND INFECTIONS 1*

### Curettage of tumour lesions of the pelvis and sacrum with the aid of cryosurgery for blood sparing and adjuvant effect

G. Scoccianti*, D.A. Campanacci, G. Beltrami, F. Frenos, R. Capanna

Ortopedia Oncologica, AOU Careggi (Florence, IT)

**Introduction** Intralesional curettage of benign aggressive tumors or metastatic lesions in the pelvis and sacrum can be a challenging procedure when dealing with wide lesions or with tumors at high risk for massive bleeding. To reduce bleeding, we began to use cryosurgery as an aid during removal of the lesion, which is progressively freezed and curetted, and as an adjuvant treatment after curettage on the remaining walls of the cavity. A retrospective evaluation of 10 cases was performed to verify efficacy and safeness of this technique.
**Materials and methods** In the last 5 years we performed 10 surgical procedures of intralesional excision of bone tumors in the sacrum and pelvis with the intraoperative aid of cryosurgery. Histology of the tumour was: metastatic lesion from renal cell adenocarcinoma in 4 cases and from hemangiopericytoma in 1, giant cell tumour 1, aneurysmal bone cyst 1, schwannoma 1, chondrosarcoma 2 cases. Location of the tumour was: sacrum 3 cases, sacroiliac area 2, periacetabular 3, ileopubic ramus 1, ischium 1. Age of the patients ranged from 19 to 77 years (average 56). Freezing was performed using cryoprobes; size and number of probes varied according to characteristics of the lesion. The defect was filled with cement or left empty. Preoperative selective arterial embolization was used in 7 cases.
**Results** Average duration of surgery was 3.6 h (2.15–6). Cumulative number (intraoperative and postoperative) of blood transfusions per patient ranged from 0 to 15 (average 3.8). In one patient control of bleeding was particularly difficult and the patient received 15 blood units; in the remaining 9 patients average number of blood transfusions was 2.5. No skin necrosis was encountered. No deep infection and no neurovascular damage occurred. Follow-up ranged from 1 to 47 months (average 19.9). Two patients affected by metastatic disease died 14 and 15 months after surgery for systemic disease. In the 7 patients with follow-up longer than 1 year no local recurrence or progression was observed.
**Discussion** In our experience, use of cryoprobes to freeze tumoral tissue in high risk lesions resulted in limited bleeding. The absence of complications showed the safeness of the technique. Effect of cryosurgery on surgical margins in intra-lesional excisions needs a longer follow-up and wider series to be evaluated.
**Conclusions** Cryosurgery as an aid during curettage of bone tumors of the sacrum and pelvis is a useful tool to decrease bleeding in a high-risk surgery.

### Complication of radiation therapy for soft tissue sarcomas: experience of Orthopaedic Oncology Department of Florence

G. Caff*, G. Beltrami, F. Frenos, G. Scoccianti, D. Matera, D. Campanacci, R. Capanna

Ortopedia Oncologica e Chirurgia Ricostruttiva, Ospedale Careggi, CTO (Florence, IT)

**Introduction** Radiation therapy (RT) has an essential role in treatment of soft tissue sarcomas (STS) together with surgery and chemotherapy. STS show significant pathological responses to radiation therapy in the form of hyaline fibrosis, necrosis and granulation tissue; despite the regression of the tumour, many side effects involving bone, soft tissue, wound, nerves, vessels, and lymphatic are associated with RT. We reviewed our cases of STS treated in our Center on the last twenty year and subjected to radiation therapy focusing on the eventual complications.
**Materials and methods** At Department of Orthopaedic Oncology of CTO, Florence, we have treated, from January 1989 to December 2009, 1055 patients affected by STS of limbs and superficial trunks. All the patients have been operated and followed by the same equip of surgeons, radiotherapist, micro-surgeons and pathologists, everybody practice in soft tissue sarcoma cases. Adjuvant protocols of RT were associated to surgery in 477 cases (45.2 %): 87 were low grade sarcoma and 390 were high grade. Chemotherapy was also associated in 180 (37.7 %) patients: 8 low grade and 172 high grade. The RT medium dose was 60 GY (range 50 to 75 GY). Side effects after Radiation Therapy have been analysed on the overall group and a statistical analysis have been performed. All minor or major complication related to RT were considered and in particular a free event survival curve regarding the two most common complications (fracture and wound dehiscence) have been performed.
**Results** At an average follow up of 7 years (1–20 yy), 78 complications occurred in the whole group (16.3 %): 17 in patients affected by a low grade STS and 61 by an high grade. The most frequent complications were: 17 cases of fractures (21.8 %), 27 wound dehiscences (34.6 %), 7 necroses (8.9 %) (interestingly, the bone in 4 cases and the flap in 3 cases), 6 neuropalsy (7.6 %), 4 lymphedemas (5.1 %) and 1 major infection. 7 patients were amputated for complication of RT.
**Discussion** Even though the important complications' rate, Radiation therapy plays an essential role in treatment of soft tissue sarcoma as well as adjuvant or neo adjuvant to surgery.
**Conclusions** A multidisciplinary approach composed by specialists with practice is always necessary to select patients affected by a STS for a possible radiation therapy.

### Surgical techniques and adjuvants in femoral solitary bone metastasis

M.A. Rosa*[1], G. Maccauro[2], M.S. Spinelli[2], C. Graci[2], S. Barreca[1], F.R. Campo[1], C. Fabbriciani[2]

[1]Scuola di Specializzazione in Ortopedia e Traumatologia, Sezione di Ortopedia e Traumatologia, Dipartimento Specialità Chirurgiche, Università degli Studi di Messina (Messina, IT);
[2]Dipartimento Specialità Ortopediche e Traumatologiche, Università Cattolica del Sacro Cuore (Rome, IT)

**Introduction** It is known as the treatment of metastatic bone lesions of the femur is affected by problems either biological, linked to the tumour histotype, as mechanical, due to the reduction of the resistance of the femur strongly subjected to stresses due to load.
**Materials and methods** The authors analyze the clinical and radiographic results of 59 femoral solitary metastases observed and treated in 10 years between January 2002 and December 2011. The metaphyseal (30 cases) and diaphyseal (29 cases) localized metastases were taken into consideration. The primary tumours were analyzed: 23 lung (17 cancer-small cell carcinoma, 6 epidermoid), 25 breast (18 hormone-dependent cancer, 7 non-hormone-dependent), 6 Kidney, 5 thyroid. The follow-up was performed at 1 and 6 months on all patients regardless of histological type. In cases of epidermoid carcinoma of lung, hormone-dependent breast carcinoma, follicular carcinoma of the thyroid and renal adenocarcinoma, which had the greater Survival, the follow-up was extended to 12, 24 and 60 months. In the 20 cases with survival of more than 60 months follow-up is been extended to 72, 84 and 96 months.

**Results** The authors have considered the functional recovery of patients in relation to employed surgical methods. The prosthetic implant has allowed an earlier resumption of activities of daily living compared to the intramedullary nail.

**Discussion** The histological type strongly conditioned the surgical treatment. The prognosis "*quoad vitam*" of tumours studied is different, in metastases from lung epidermoid carcinoma, hormone-dependent breast cancer, renal cell carcinoma and follicular carcinoma of the thyroid, was necessary extensive surgery, with en bloc resection and reconstruction with tumoral prosthesis for metaphyseal localization, while for the diaphyseal locations the technique has provided Locked nailing + cement and/or intercalary prosthesis. In cases where the survival is less than 1 year, even if the bone lesion is solitary, as in metastatic breast cancer and non-hormone-dependent small cell carcinoma of the lung, orthopaedic surgery was based on femoral nail with long locked PFN in 9 cases, with long nail locked ENDOVIS + cement in 15 cases. Surgery of metastases, where possible, was always associated with the surgical treatment of primary tumour at the same time.

**Conclusions** The surgical treatment was always associated with radiation treatment, replaced by radio-iodine therapy for thyroid, secondary lesions; chemotherapy and hormonal therapy followed a uniform protocol across different centers, but different according to the histological type.

### *C24—HIP 4*

### Periprosthetic hip femoral fractures: treatment issues

E. Betti*, F. Gambini, E. Gori, G. Guido

Clinica Ortopedica, Azienda Ospedaliera Universitaria di Pisa II (Pisa, IT)

**Introduction** Periprosthetic hip femoral fractures are a technically challenging problem due to increasing incidence and their polymorphism. Usually the fracture site, the stability of femoral stem leads the choice of the treatment between reduction with internal fixation and revision arthroplasty (Vancouver Classification system [1], but often the serious comorbidities and low life expectancy in elderly patients strongly influences the decision-making process [2]. In these patients who are unfit for more prolonged surgical procedures, the treatment is forcedly limited to the primary stabilization of the fracture also when the prosthesis is loose.

**Materials and methods** We reviewed a consecutive series of 18 patients with periprosthetic hip fractures treated with cable-ready plate system: 15 female and 3 male, mean age 81 years (min. 73-max. 100). These fractures were classified according to the Vancouver Classification system: 4 were type B1, 11 type B2–B3 and 3 type C. 4 fractures type B1 and 3 type C (prosthesis stable) were treated with open reduction and internal fixation. 4 fractures type B2-B3 (prosthesis loose) with a long-stemmed revision and additional internal fixation. In 7 elderly patients with fractures type B2-B3 the treatment was limited to a primary stabilization of the fracture by an internal fixation.

**Results** We could assess the clinical and radiographic outcomes of 12 patients after 6 months (4 patients died, 2 were too frail to attend follow-up). 8 patients showed bone union and a good functional outcome: 3 fractures B2-B3 treated with revision arthroplasty and osteosynthesis and 5 B1 and C fractures treated with ostheosyntesis. 4 elderly patients, fracture B2-B3 with stem loosening, where we were forced by their serious general condition to perform the simple internal fixation, continued to have the primary stability of the fracture.

**Discussion** The algorithm of Vancouver Classification is an extremely reliable frame of reference in periprosthetic fracture treatment, but we cannot always follow it strictly in elderly patients with low life expectancy for whom we are obliged, even with loose prosthesis, to carry out more simple surgical procedure of osteosynthesis since they are unable to undergo more complex procedure such as revision arthroplasty.

**Conclusions** Valid primary stabilization of periprosthetic fracture given by reliable synthesis techniques, such as the cable ready system, is fundamental in the treatment strategy both where it is crucial to the fracture consolidation process in which we have been able to follow correct biomechanical reference criteria and where, in patients whose general condition is precarious, the stable fixation of the fracture is an essential factor in their survival.

**References**

1. Duncan CP, Masri BA (1995) Fractures of the femur after hip replacement. Instr Course L1995 44:293–304
2. Parvizi J, Vegari DN (2011) Periprosthetic proximal femur fractures: current concepts. J Orthop Trauma 25(Suppl 2):S77–S81

### Pseudotumour occurring in total hip arthroplasty with metal on metal bearing and small diameter head

P. Piciocco*[1], N. Santori[2], F. Falez[3], F.S. Santori[2]

[1]Policlinico Umberto I (Rome, IT);
[2]Rome American Hospital (Rome, IT);
[3]Santo Spirito in Sassia (Rome, IT)

**Introduction** The metal on metal bearing in hip prosthesis has been developed to extend the longevity of the implant and to ensure a greater range of motion to the patient by increasing diameter of the heads. With the longer follow-up have emerged problems regarding the release of nickel, chromium and cobalt, ions including the growth of pseudotumour. These problems seem to be an exclusive of large diameter head and they have not been described in the small and medium ones. Purpose of this paper is to describe the cases of pseudotumor occurred in 3 patients with small-diameter heads.

**Materials and methods** 156 hip arthroplasty with metal-on-metal bearing with head 28, 32, 36 and 38 mm were implanted in 132 patients between 1996 and 2004. The mean follow-up was of 12.4 years. In 34 cases (the group enclosed the cases with pseudotumour) are measured blood values of chromium, cobalt and nickel.

**Results** Of the 132 patients 17 were lost at follow-up, 2 patients underwent a revision for deep infection, 2 have reviewed the acetabular component for aseptic loosening and 1 case had late dislocation. In 3 cases, finally, a pseudotumour was observed at 12, 11 and 10-year follow-up respectively.

**Discussion** The pseudotumour is non-septic inflammatory lesions occurring in the soft tissues near the implant. In most cases they are completely asymptomatic. In others these masses tend to grow and the symptoms depend to compression of surrounding structures. We discuss the pathogenesis of the lesion. The growth does not seem strictly linked to increased wear or higher blood levels of metal ions.

**Conclusions** This study shows that the development of pseudotumour is not an exclusive of the large diameter heads but it seems to be probably linked to an individual predisposition of the patient.

### Thigh pain incidence at 24 months after total hip arthroplasty with the short stem Tri-Lock BPS, Gription coating: a prospective study

M. Ulivi*[1], L. Orlandini[1], M. Bassi[1], V. Meroni[1], V. Sansone[2]

[1]Unità Operativa di Ortopedia e Traumatologia, Istituti Clinici Zucchi (Monza, IT);
[2]Clinica Ortopedica dell'Università degli Studi di Milano, Istituto Ortopedico Galeazzi IRCCS (Milan, IT)

**Introduction** Thigh pain incidence after cementless total hip arthroplasty in literature may vary from 1.9 % to 40.4 %. Thigh pain incidence with the original Tri-Lock design varies from 2 to 9 %. The short stem Tri-Lock (Tri-Lock BPS) which has been reduced in dimension and geometry in comparison to the original Tri-Lock, is available in the US since 2009 and in Europe since 2010. The new surface treatment employs the Gription TM DePuy coating, (a complex 3D matrix highly porous and rough).Aim of the study is to evaluate if the new stem version is able to reduce thigh pain incidence, confirming the indication in young and active subjects.
**Materials and methods** All patients have been consecutively operated by the same surgeon from March 201 to date. The study is still ongoing and to date 111 consecutive patients received the cementless Tri-Lock BPS stem, Pinnacle cup, Marathon polyethylene liner (DePuy) and ceramic head. Patients are prospectively followed up for a period of 24 months. Postoperative evaluation is done with a visual analogue scale (VAS) including pain mapping and the Harris Hip Score. Clinical and radiological follow up is at 6-12 e 24 months from surgery. For thigh pain is utilized the Barrack definition: only pain reported in the anterior aspect of the thigh and distal to the inguinal ligament is considered significant.
**Results** We report preliminary results of the first 70 patients enrolled in the study (20 males, 50 females, mean age 66.09) whose follow up at 12 months has so far been completed. Light thigh pain (mean VAS value 4.33) has been reported in 3 patients (4.2 %) No correlations of thigh pain with clinical or stem related variables could be detected.
**Discussion** The 4.2 % light thigh pain at short term is favourable if compared to the incidence of thigh pain of the original design which has shown thigh pain incidence values of 9 % at long term.
**Conclusions** Preliminary data suggest that Tri-lock BPS seems indeed able to reduce the incidence of thigh pain and favours functional rehabilitation. Gription porous coating allows an adequate primary fixation which is very important in young and active patients.

### Post-prosthetic dislocation prevention in total hip revision surgery using a dual mobility design

R. Philippot[1], M. Stamilla*[2], F. Farizon[1], G. Gresta[1]

[1](Saint Etienne);
[2](Catania, IT)

**Introduction** Many publications have already shown the great interest of dual-mobility concept which significantly reduces the rate of prosthetic dislocation and thus find its place for patients at high risk of post operative instability. The aim of our study is to evaluate the prevalence of prosthetic instability in revision total hip arthroplasty using a dual-mobility cup.
**Materials and methods** Our multicentric series consists of 163 revision total hip arthroplasties performed between May 1999 and May 2004. The mean age at revision was 68,7 years (34-92) and the mean follow-up period was 56 months. The primary aetiology necessitating revision is aseptic loosening. According to the SOFCOT classification, the acetabular deficiency was grade IV 9 times, grade III 47 times, grade I or II 107 times. All the implanted acetabular components are SERF dual-mobility implants. This system consists of a metal back which can be HA-coated and Press Fit or cemented in a Kerboull cross or in a Novae Arm. The mobile-bearing insert which allows a dual articulation between the head and the metal back is of polyethylene. We implanted 119 HA-coated press-fit cups and cemented 44 dual-mobility cups in a support ring or in a Kerboull cross.
**Results** The mean Merle d'Aubigné and Postel score is 14.1 at the last follow-up and 4.2 in the preoperative period. We reported 8 complications: 6 early dislocations and 2 acetabular revisions for secondary mobilisation of the cup.
**Discussion** According to Huten's 1996 SOFCOT teaching conference, this rate ranges between 2 and 5 % and we have already published a series of 106 dual-mobilities with no dislocation at a 10-year follow-up period. With 4 % dislocations at a mean follow-up of 56 months, the dual-mobility cup seems to provide high stability in revision hip surgery when other factors such as muscular deficiency, extended synovectomies, difficult implant placement, encourage an uncertain post-operative prosthetic stability. These results have to be compared to those of other systems such as constrained acetabular cups or tripolar cups.
**Conclusions** This is why dual-mobility remains an efficient and reliable choice to avoid prosthetic dislocation in revision surgery. Moreover, we encourage the use of dual-mobility cup in any high risk situation in terms of post-operative instability such as for old or neurological patients.

### *C25—PAINFUL PROSTHESES 2*

### Ankle replacement revision: a new algorithm of treatment

F.G. Usuelli*[1], M. Boga[1], M. Grassi[2], U. Alfieri Montrasio[1]

[1]CTS Piede e Caviglia, IRCCS Galeazzi (Milan, IT);
[2]Università degli Studi di Milano (Milan, IT)

**Introduction** With the recent improvement of ankle replacement, a new interest in this surgical technique is growing up. This also means new problems such the need to revise the failures of this type of surgery.
**Materials and methods** Clinical (AOFAS score) and radiographic data (Weight bearing X-rays + Saltzman views) were collected in 32 consecutive patients of our Ankle Replacement Cinics (CTS Piede e Caviglia). Inclusions criteria were: a preview ankle replacement performed at least 6 months before, AOFAS score < 50, patient unsatisfaction. The patients were all divided in three groups. GROUP A (n = 20): pain referred to other joint around the ankle. GROUP B (n = 10): pain at ankle joint with no sign of component subsidence neither mobilization. GROUP C (n = 2): pain in the ankle with mobilization of the replacement. GROUP A: treatment were focused of realignment (two double fusions and 18 prescription of orthosis with good results at 6 months from prescription. GROUP B: revision of both components with good results at 6 months from surgery tIme. GROUP C: TTC fusion with cadaveric femoral head graft.
**Results** Good results at 6 months (AOFAS score > 60).
**Discussion** In all those cases with maligment or component mobilization it is mandatory a surgical choice. In all the other cases we suggest a conservative treatment.
**Conclusions** A good imaging study is necessary not only for preoperative planning but also to understand the function or dysfunction of these patients and to treat them properly.

### Thigh pain after total hip arthroplasty

L. Pierannunzii*, A. Guarino

Istituto Ortopedico Gaetano Pini (Milan, IT)

**Introduction** Thigh pain (TP) in THA is usually associated with stem loosening. When the stem is proven to be fixed, its causes are still unclear. Thus a wide case series was analyzed to point out TP-related clinical and radiological features of several types of femoral component design.
**Materials and methods** 325 patients were evaluated 21 months on average (from 4 to 59) after uncemented total hip replacement. Stems were divided into 4 groups: extensively porous and squared-section, proximally coated and conical, proximally coated and anatomical, neck preserving. TP was diagnosed when patients complained about a load-related mid-thigh anterolateral pain, neither irradiated to the groin nor to the buttock, without infection signs. All positive cases were analyzed according to the classification published by the author [1] and quantified with VAS.
**Results** Thirty-one patients of 325 reported TP, with VAS equal to $36 \pm 9$. The distribution varied significantly with stem design, from the maximum of 22 % in group III (proximally coated anatomical stems) to the minimum of 4.5 % in group I (extensively porous and squared-section). The physiopathological classification varied among the groups: in group I dynamic TP from macroinstability (i.e., loosening) prevailed; in group II both forms of dynamic TP, from macro- and micro-instability (i.e. fibrous fixation), were equally represented; in group III dynamic TP from microinstability prevailed; in group IV static TP from distal fixation was the most common.
**Discussion** This analysis shows that TP is still present with modern THA, although symptoms are usually mild and well tolerated. As a matter of fact, only loose stems required surgery. Anatomical stems are at most risk, especially if canal filling is inadequate and the fixation is mainly fibrous. On the other side, wedge-like stems seem to be less prone to TP, regardless of the bone filling. The study has some limitations, such as the heterogeneity of the prosthetic designs and the different size of the four groups.
**Conclusions** Prevalence of TP varies with stem design. A careful stem selection and an appropriate implantation technique are mandatory to prevent it, peculiarly if less tolerant designs are chosen (anatomical or neck-preserving).
**Reference**
1. Pierannunzii LM (2008) Orthopedics 31(7):691–699

### Modular and tantalum augmentation to treat type 2-3 bone defects

M. Vasso*[1], S. Cerciello[1], A. Hassan[2], A. Schiavone Panni[1]

[1]Università del Molise (Campobasso, IT);
[2]Zimmer GmbH (Winterthur, DE)

**Introduction** Controversy exists about the best management for a durable reconstruction of bone defects in revision total knee arthroplasty. The purpose of this study was to determine if use of modular augmentation to reconstruct type 2 and 3 defects significantly increased clinical and functional outcomes, caused radiolucency or osteolysis, affected mid-term survivorship of knee joint reconstruction.
**Materials and methods** Thirty-eight consecutive revision knee arthroplasties were performed at our institution. The 38 patients were followed for a mean follow-up period of 7 years. Type 2 and 3 defects were treated with metal augments, tantalum cones, and modular cementless stems. In particular, tantalum cones were used only for management of type 3 defects. Patients were assessed using the IKS knee and function scores, and the HSS score. Mean average knee flexion was reported. Radiographic evaluation was completed for all 38 patients.
**Results** The average IKS knee and function scores, HSS score, and knee flexion significantly improved at the final follow-up. All metal augments and tantalum cones appeared well fixed radiographically. Radiolucencies (tibial, incomplete and not progressive) were observed only in 2 cases. Two femora presented minimal osteolysis. Re-revision was necessary in three (7.9 %) patients, because of causes independent of use of modular augmentation.
**Discussion** The greatest challenge during revision total knee arthroplasty is certainly bone loss, which could primarily affect final functionality and survivorship of the knee joint reconstruction. The management of bony defects in knee revisions is still controversial because of the variety of defects encountered and the relative lack of evidence from clinical trails or experimental studies. Modular augmentation certainly represents an optimal solution for the management of severe bone loss because its extensive modularity, quick and easy use, and large availability.
**Conclusions** Modular augmentation may reduce the need for bone grafting or allografting to treat type 2 and 3 bone defects, providing a well-functioning and durable knee joint reconstruction.

### Heterotopic ossification (HO) after prosthesis-based hip replacement

P.V. Frediani*, A. Pascazio, D. Baù

Ospedale S. Antonio Abate (Gallarate, IT)

**Introduction** Heterotopic ossification (HO) is characterized by mature and medullary lamellar bone in periarticular connective tissue. HO is a frequent complication after hip prosthesis application. Aetiology and predisposing factors are not fully known, although some possible causes have been identified: hypertrophic oa; post-traumatic arthrosis with osteophytosis; male gender; surgery complications such as soft tissue traumatism; some metabolic forms.
**Materials and methods** Our work describes statistical incidence based on experience. Lesion physiopathology is analyzed. Focus is put on the importance of lab. tests, bone scintigraphy and C.A.T. scan in view of an early diagnosis and assessment of calcification maturity. Prevention and treatment therapy suggestions are discussed.
**Results** Our case study shows a 40 % incidence for calcifications after 1 year from hip prosthesis implant. Only 12 % of these had clinical implications such as pain, worsened articular elasticity, anchylosis. Our results are within the average of published case studies. Exeresis surgery treatment is limited to frankly symptomatic cases of mature calcifications.
**Discussion** Main issues faced by surgeon in case of such a complication are: prevention, early diagnosis, treatment. Main prevention therapies are: a fans-based therapy immediately after surgery and an early articular mobilization, especially for vulnerable patients. Early diagnosis is based on monitoring alcaline phosphatase and urinary PGE's. Bone scintigraphy is specially sensitive, allowing a diagnosis after 2.5 weeks from surgery. C.A.T. scan is useful for an early diagnosis, identifying calcifications 4–6 weeks before radiography and allowing assessment of calcification maturity. Treatment details and timing are referred to frankly symptomatic patients, in the presence of mature calcifications.
**Conclusions** Periprosthetic calcifications still represent an unsettled issue. From a physiopathologic point of view, a morphogenetic protein may be responsible for calcifications, once it is released from normal bone during surgery. Prevention is multi-factorial, based on prophylaxis with fans (cox2, indomethacin) for at least 7 days in vulnerable individuals; on a non-traumatic surgical technique; on early articular mobilization. Monitoring alcaline phosphatase and urinary PGE's, bone scintigraphy and C.A.T. scan enable an early diagnosis as well as assessment of calcification maturity. Surgical therapy is statistically limited to few frankly symptomatic cases, only in the presence of mature calcifications. Radiotherapy is applicable when calcifications appear, at the stage when they are not yet mature.

### *C26—SHOULDER AND ELBOW 6*

### Anatomical features of the suprascapular notch: correlation with scapular dimensions and clinical relevance

P. Albino*, V. Arceri, D. Passaretti, V. Candela, S. Carbone, S. Gumina

Dipartimento di Scienze dell'Apparato Locomotore, Università di Roma "Sapienza" (Rome, IT)

**Introduction** An improved knowledge of the suprascapular notch anatomy may help to prevent and to well-assess the suprascapular nerve entrapment syndrome. We aimed to verify the reliability of the existing data, to assess the differences between the two genders, to verify the existing correlation between the dimensions of the scapula and of the suprascapular notch, to investigate the relationship between the suprascapular notch and the postero-superior limit of the safe zone for the suprascapular nerve.
**Materials and methods** We examined 500 dried scapulae, measuring seven distances referring to the scapular body and suprascapular notch; they were also catalogued according to gender, age and side. Suprascapular notch was classified according to Rengachary's method. We elaborated for each class the width/depth ratio. Pearson's correlation was also calculated.
**Results** Frequencies were: Type I 12.4 %, Type II 19.8 %, Type III 22.8 %, Type IV 31.1 %, Type V 10.2 %, Type VI 3.6 %. Width and depth showed a not statistical significant difference when elaborated according to gender and side; a significant difference was found between the depth means elaborated according to median age (73 years old). Correlation indexes were weak or not statistically significant. The difference between the postero-superior limit of the safe zone in the six types of notches was not statistical significant.
**Discussion** We observed a difference concerning the frequencies of the VI Types of suprascapular notch presented in our study respect to those reported in literature. We suppose that the difference between the depth means may be due to the most frequent presence of partial/total ossification of the superior transverse scapular ligament in the population over 73 years old. The characteristics of the suprascapular notch (dimensions and type) are not influenced by age, gender and scapular dimensions.
**Conclusions** A pre-operative evaluation of the patient's characteristics (gender, age and scapular dimensions) does not provide any information about the suprascapular notch. The safe zone is not influenced by the morphologic and morphometric characteristics of the suprascapular notch.

### Pigmented villonodular synovitis of the shoulder associated with massive rotator cuff tear treated with arthroscopic synovectomy and debridement

S. Gumina*[1], S. Carbone[1], M. Borroni[2], V. Campagna[3], D. Passeretti[1], A. Castagna[2], F. Postacchini[1]

[1]Ortopedia e Traumatologia, Università di Roma Sapienza (Rome, IT);
[2]Istituto Clinico Humanitas (Milan, IT);
[3]Policlinico Militare Coelio (Rome, IT)

**Introduction** Pigmented villonodular synovitis (PVNS) is a rare condition. We present the largest series of patients with shoulder PVNS, massive irreparable rotator cuff tear and gleno-humeral osteoarthritis treated with arthroscopic debridement and synovectomy.
**Materials and methods** We treated 9 patients (6F and 3 M, mean age 65.8 years, range 63–70 years) with PVNS of the shoulder; irreparable rotator cuff tear and slight gleno-humeral arthropathy (group I). Patients underwent arthroscopic synovectomy and debridement. Results were compared with those obtained from a control group (group II) made up of 33 consecutive patients (21F and 12 M) who underwent arthroscopic debridement for irreparable cuff tear and had no (23 patients) or slight (10 patients) gleno-humeral arthropathy (Constant Score).
**Results** The preoperative shoulder function in group I was reduced with respect to group II (CS: 12 *vs*. 35). Upon follow-up the CS value was lower in group I than in group II (CS: 40 *vs*. 54). In the preoperative period, the differences between the two groups relating to the CS value and to each item of the score were always statistically significant; instead, upon follow-up, significant differences emerged in the CS value, ADL and ROM. Differences relative to pre-operative CS values in group I and II were statistically significant; while, at the follow-up period, significant differences emerged only between CS values of patients with PVNS and the values of group II without gleno-humeral arthropathy. No differences emerged between pre and post-operative CS values in group II without or with gleno-humeral arthropathy.
**Discussion** PVNS of the shoulder is a rare condition. Recent literature refers on sporadic case reports which affected above all middle aged or elderly patients. Only two papers described respectively of one case of PVNS of the shoulder that occurred in two adolescent males.
**Conclusions** Arthroscopic synovectomy is an effective surgical treatment for PVNS; the poor functional outcome can be attributed to the coexistence of the gleno-humeral arthropathy. Three fourths of patients with PVNS had shoulder osteoarthritis; this percentage cannot be simply attributed to natural history of massive irreparable cuff tears; but a direct role of synovitis has to be considered.
**Level of evidence** Level IV.

### The "double pulley" technique for arthroscopic fixation of partial articular-side bony avulsion of the supraspinatus tendon: a rare case of "bony PASTA" lesion

L. Murena*[1], G. Canton[1], D. Falvo[1], E. Genovese[2], M. Surace[1], P. Cherubino[1]

[1]Dipartimento di Scienze Chirurgiche Ricostruttive e Tecnologie Avanzate, Università degli Studi dell'Insubria (Varese, IT);
[2]Dipartimento di Scienze Chirurgiche e Morfologiche, Università degli Studi dell'Insubria (Varese, IT)

**Introduction** The "bony PASTA" lesion is a partial articular-side bony avulsion of the supraspinatus tendon. It was described only once up to date in literature in 2007 by Bathia et al. These authors presented an arthroscopic technique for fixation of the avulsed fragment using a single medial suture anchor with simple mattress sutures. We present the use of the double pulley technique for arthroscopical fixation of bony PASTA lesions.
**Materials and methods** We report the case of a 22 years old man presenting with a bony PASTA lesion to the right shoulder following a motorbike accident. The lesion was documented with X-rays and MRI exams. The surgical technique involved the use of a 30° and 70° arthroscope. Intraarticular observation documented a 15 long and 5 mm wide avulsion with two principal fragments. Subacromial observation documented the integrity of the lateral insertion of the supraspinatus tendon to the greater tuberosity. Two double-loaded suture anchors were implanted through the lateral intact aspect of the supraspinatus tendon with a transtendinous technique. The anchors were placed at the anterior and posterior extremities of the bony lesion respecting the tendon insertion to the avulsed fragment. Each of the 4 medial sutures was retrieved in the subacromial space through the intact supraspinatus tendon medial to the fracture. Reduction and fixation of the avulsed fragment was initially obtained with a simple suture for each anchor oriented from medial to lateral. The remaining suture wires were coupled in double pulley configuration generating two sutures oriented from anterior to posterior.

**Results** At the end of the procedure adequacy of reduction was confirmed by intra-articular arthroscopic observation throughout complete range of joint motion. At 2 months from surgery the patients fully recovered daylife activities and X-rays and arthro-MRI imaging documented healing of the fracture.

**Discussion** The advantage of the double-pulley configuration is the compression of the osteo-tendinous complex obtained along the whole anterior-posterior length of the lesion, unreachable with medio-lateral oriented simple sutures. Moreover double pulley configuration creates a waterproof reduction of the fragment, protecting the fracture site from the negative effect of sinovial fluid on bone healing.

**Conclusions** In our opinion the double pulley technique allows optimal reduction of bony fragments and reconstruction of normal footprint anatomy even in comminuted fractures.

### Non-operative management of shoulder adhesive capsulitis: effectiveness of the Lyon hydrotherapy rehabilitation program

F. Cautiero*[1], R. Russo[1], G. Della Rotonda[2], F.P. Di Domenico[3]

[1]U.O.C. di Ortopedia e Traumatologia, Ospedale dei Pellegrini (Naples, IT);
[2]Scuola di Specializzazione in Ortopedia, Università Federico II (Naples, IT);
[3]Centro di Riabilitazione “Minerva” (Naples, IT)

**Introduction** We evaluated the mid-term efficacy of the Lyon hydrotherapy rehabilitation program in the treatment of adhesive capsulitis.

**Materials and methods** From January 2000 to January 2005, we treated 250 patients with adhesive capsulitis using hydrotherapy rehabilitation according to the Lyon program. The patients were treated for an average of 9 weeks (27 sessions). 180/250 patients (72 %) were re-examined at a minimum of 12 months (range 12–108 months) using the modified Constant score (n = 100) and the VAS scale (n = 100) or the Simple Shoulder Test-SST (n = 80).

**Results** The mean modified Constant score was 82.6 (range 54–90), the mean VAS score was 8.6 (range 2–10) and the mean SST was 10.5 (range 8–12). 10/180 patients (0.5 %) were treated with arthroscopic release.

**Discussion** The Lyon hydrotherapy rehabilitation program gives good mid-term results as regards objective shoulder scores, range of motion and patient satisfaction. Consequently, this non-operative intervention may be considered for patients affected by adhesive capsulitis.

**Conclusions** The Lyon hydrotherapy rehabilitation program results in good outcome about pain and range of motion. The mid-term results regarding objective shoulder scores and patient satisfaction are high. The failure rate was low, so this non-operative intervention may be considered for patients affected by adhesive capsulitis.

### *C27—SPINE 5*

### Indication to occipito-cervical fusion in upper cervical spine instability

M. Cappuccio*, S. Paderni, G. Bosco, G. Scimeca, L. Mirabile, L. Amendola, F. De Iure

UOSD Chirurgia Vertebrale, Ospedale Maggiore (Bologna, IT)

**Introduction** Occipito-cervical (OC) fusion and instrumentation is indicated for a variety of conditions with OC instability. The causes of OC instability include trauma, inflammatory disorders, infection, tumours, iatrogenic decompression, and congenital anomalies. The indication for OC fusion in trauma patients, an extensive posterior instrumentation fixation procedure that sacrifices the motion of the occipital and C1–2 complex and a variable amount of subaxial motility, is still controversial.

**Materials and methods** In a clinical retrospective study, the authors reviewed clinical and radiographic results of occipito-cervical fusion in 12 patients with upper cervical spine trauma, and 5 patients with cranio-cervical junction abnormalities, including atlanto-axial instability and progressive basilar invagination.

**Results** The average surgical time was 190 min (range 130 to 270 min). Postoperative analgesia was performed in all cases with a 36 h lasting elastomeric pump containing an opioid and an NSAID. All the patients were granted an immediate mobilization in the bed with a soft collar. Two elderly patients died of pneumonia after 6 and 8 days in ICU. No surviving patient had neurological deterioration postoperatively. All patients with basilar invagination improve the neurological function, measured with the scale of Nurick, and the radiographic parameters.

**Discussion** OC fusion is a surgical procedure that affects almost the entire normal range of motion of the head. It should be so reserved in selected cases of instability of the cranio-cervical junction causing neurological deficits or potential serious neurological damage. In the field of traumatology absolute indication for OC arthrodesis is the atlanto-occipital dissociation. In cases of basilar invagination is a safe operation, with good clinical and radiographic results.

### Results at three years following the implant of interspinous spacers (X-STOP and BAC-JAC PIONEER) for the treatment of neurogenic claudication caused by lumbar stenosis: our experience of 263 cases conducted between December 2007 and December 2011

F. Bove*[1], G. Bove[2], G. Bove[2], G. Fabiani[1], F. Volpato[1]

[1]Istituto Neurotraumatologico Italiano (Grottaferrata, Rome, IT);
[2]Università Sapienza (Rome, IT)

**Introduction** Backache is an extremely common pathology. Every year in the United States alone medical costs exceed $100 billion and specialists perform more than 15 million examinations. Approximately 4 % of patients suffering from chronic backache are affected by neurogenic claudication caused by lumbar stenosis. Currently the most common conservative treatments for lumbar stenosis are physiotherapy, rest, medical therapy, local corticosteroid infiltrations; invasive surgical treatments range from laminectomy to stabilisation with means of synthesis. Interspinous spacers, already described in the 1950 s, represent a valid alternative for the treatment of lumbar stenosis. The current, technically improved, devices have a high degree of safety and reliability, increasing the interest in their application.

**Materials and methods** The aim of this work is to describe our experience with the 263 cases treated between December 2007 and December 2011. Patients were assessed using the VAS scoring system and a questionnaire that considered functionality, pain and posture.

**Results** The survey involved a total of 109 patients. The majority of cases displayed a good level of tolerability to the device, with a degree of satisfaction and solution of the symptomatology of around 80 %.

**Discussion** Our results are consistent with similar studies conducted on European populations.

**Conclusions** The interspinous spacers are not a substitute for surgical decompression for those patients suffering from severe lumbar stenosis, but they represent a safe, effective and less invasive alternative for a well selected group of patients affected by neurogenic claudication.

### Proposal for a study protocol on the effectiveness of biophysical stimulation in bone fractures by skeletal fragility

C. Ruosi*, S. Liccardo, D. Rossi, F. Granata, A. Barbato, G. Colella, S. Lupoli

Dipartimento di Ortopedia e Traumatologia, Università degli Studi di Napoli "Federico II" (Naples, IT)

**Introduction** Biophysical stimulation has been used for several years in the orthopaedic field to promote post-traumatic fracture healing and spinal fusion. It is also described in the literature a beneficial effect on pain and a reduction in the use of NSAIDs. We analyzed the effect of biophysical stimulation in controlling pain in patients with osteoporotic vertebral fractures and proposed a protocol of study for the evaluation of its effectiveness in reducing the consequences of osteoporotic vertebral collapses.

**Materials and methods** We considered several pre-clinical and clinical studies concerning the use of physical stimulation (pulsed electromagnetic fields and electric fields) in the treatment of vertebral fragility fractures.

**Results** A study of Massari et al. performed on 42 patients treated with lumbar or lumbo-sacral fusion because of traumatic vertebral fractures or spinal degenerative disease, showed a positive effect of physical stimulation on pain reduction. A Rossini's et al. prospective, randomized, double-blind study showed a positive effect of physical stimulation on pain reduction and a dose–response effect, as well as a reduction in the use of analgesics in women with multiple vertebral fractures. Moreschini et al. examined, in a group of elderly patients with osteoporotic vertebral fractures, data relating to pain, intake of NSAIDs, and the time of immobilization in a orthopaedic brace, in order to verify if the early application of CEMP would be able to improve clinical outcome in the short term. The results of this study showed a reduction of back pain, intake of NSAIDs, a faster functional recovery and deambulation without an orthopaedic brace.

**Discussion** It has been demonstrated, both in preclinical studies and in clinical trials, that the physical stimulation is able to: (1) increase cell proliferation, and thereby provide an expansion of the osteoprogenitor-cell's pool; (2) promote the synthesis of growth factors, such as TGF-ß and BMPs; (3) significantly reduce the production of inflammatory cytokines (TNF-α, IL-6, IL-1ß).

**Conclusions** We documented the efficacy of biophysical stimulation in patients with vertebral fractures caused by bone fragility. Its effect on pain results in a better functional recovery, a reduction in the intake of analgesics and in a significant improvement in the quality of life. In the end, we'll evaluate the ongoing study's results on the effects of biophysical stimulation on bone consolidation in vertebral collapse and its ability to prevent subsequent collapses at other levels.

### Percutaneous treatment of the spinal metastases

A. Zachos*[1], M. Spiga[2], C. Doria[2], P. Tranquilli Leali[1]

[1]Clinica Ortopedica, Universita' degli Studi di Sassari (Sassari, IT); [2]U.O.C Ortopedia e Traumatologia, San Martino (Oristano, IT)

**Introduction** The spinal column is the most frequent site of bone metastasis, and between 30 % and 70 % of patients with cancer will have evidence of spinal metastasis at autopsy. The majority of metastasis occurs in the thoracic spine (70 %) followed by the lumbar (20 %) and cervical region (10 %). The surgical treatment of the vertebral metastases remains a real challenge in spine surgeons. Recent advances in surgical techniques allow a less aggressive approach of the patient with better results in terms of decreasing pain, improvement of the quality of life. We must avoid the overtreatment of terminally ill patients, some patients may survive for several years and benefit from surgery.

**Materials and methods** The surgical techniques that we used from August 2006 to October 2011 were the coablation associated with vertebroplasty and percutaneous osteosynthesis. Very important were the operating room set up and the surgical technique. In our clinic, in the last 5 years, 115 patients were treated, 76 patients (94 vertebrae) with vertebroplasty and ablation. We used the percutaneous osteosynthesis in 39 patients aged between 42 and 88 years (mean 65 years).

**Results** In both types of treatments, the postoperative elapsed were regular with early mobilization and regression of pain.

**Discussion** The diagnosis and treatment of spinal metastases require multidisciplinary review. The optimal treatment depends from a balance between the morbidity of the surgical procedure, the estimated survival time, and the overall quality of life.

**Conclusions** We believe that these minimally invasive techniques are certainly a viable alternative to ''open'' traditional spine surgery and can help in order to reduce the pain and to restore the stability.

### Efficacy and safety of the correction of adolescent idiopathic scoliosis (AIS) with pedicle screw-sublaminar wires (universal clamps) hybrid construct: description of surgical technique and preliminary results

G. Collo*[1], F. Trucchi[2], A. Maiello[3], F. Castoldi[3], P. Rossi[3]

[1]Ospedale CTO-Maria Adelaide (Turin, IT);
[2]Ospedale Mauriziano Umberto I (Turin, IT);
[3]Università degli Studi di Torino (Turin, IT)

**Introduction** The objective of this study is to describe the surgical technique with hybrid construct for surgical correction of AIS and the description of the results in 30 cases.

**Materials and methods** We included all patients underwent surgery for AIS between 2010 and 2012. Surgical procedure started with median longitudinal incision centered at the main curves. After the subperiosteal detachment of paraspinal muscles, 4 to 6 peduncolar screws are placed at the lumbar level. Then 3 to 7 sub-laminar wires (UCs) are placed on the side of concavity and one on convexity at the apex of the curves. Four UCs are placed at proximal end of instrumentation. Two correction pre-bent bars, combined with 2 or 3 transverse connectors, are solidarized with lumbar peduncolar screws and UCs. Correction was performed using posteromedial translation and derotation. Then intraoperative awakening test is performed. After the preparation of arthrodesis bed, autologous and homologous bone grafts are placed. For each patient, we evaluated the percentage of correction both of coronal and sagittal plane, operative time, blood loss, perioperative complications and hospital length of stay.
**Results** Thirty patients (25 males 5 females) underwent a surgical procedure, with a mean age of 14.5 (SD ± 5) years. In the coronal plane postoperative values were improved by 60 % (SD ± 13 %), while in the sagittal plane values were improved by 55 % (SD ± 15 %). The average operative time were 220 (SD ± 20) minutes, while perioperative blood loss have not been significant and managed only with the autotransfusion (n = 2) in 94 % of patients. There were no perioperative complications and the average time of hospitalization was 7 days (SD ± 2).
**Discussion** The objective of the surgical correction of AIS is to prevent the evolution of the scoliotic curves using a construct to obtain solid stable arthrodesis with a low number of complications. It's fundamental to obtain good correction especially in the sagittal plane, as well as in the coronal plane. The use of UCs has the advantages of the sublaminar wires technique with fewer risks of metal used in the past.
**Conclusions** Sublaminar wires technique with UCS is reliable and reproducible with excellent corrections both in the coronal plane than in the sagittal plane. It is also able to reduce the time and surgical stress for the patient.

### *C28—NEOPLASTIC DISEASES AND INFECTIONS 2*

### Knee arthrodesis with external fixation or intramedullary nail: comparison between two different surgical techniques

M. Lo Presti, F. Iacono, D. Bruni

Istituto Ortopedico Rizzoli (Bologna, IT)

**Introduction** Knee arthrodesis is certainly an option of last choice but to find space after complex cases of periprosthetic knee infection.
**Materials and methods** The objective of this study was to compare clinical and functional results of knee arthrodesis made with 2 different surgical techniques: synthesis by intramedullary nail (Link Laboratory, Hamburg, Germany) and Hoffman external fixator type II (Stryker Kalamazoo, Michigan USA). The study included 31 patients (22 treated by intramedullary nail and 9 patients with external fixator Hoffman II) who underwent arthrodesis between 2001 and 2009 as a result of prosthetic knee infection. The outcome of the procedure was assessed using the Visual Analogue Scale (VAS) and Lequesne Algofunctional Score. Moreover a clinical evaluation of patients was carried out, including data such as limb length discrepancy, complete healing of surgical wounds and radiographic evaluation to confirm presence of bony fusion, or pseudoarthrosis.
**Results** The comparison between the 2 techniques showed better VAS values in the group of patients treated with intramedullary nail (mean VAS with intramedullary nail 3 *vs.* 6 with E.F) and values comparable between the two groups with Lequesne Algofunctional Score (11.13 Nail intramedullary *vs.* FE 13.4). In addition, the residual leg length discrepancy in the two groups was statistically significant: mean 0.8 cm (range 0–2.2 cm) in the intramedullary nail compared with average 2.6 cm (1.5–4.2) in the group with external fixator. The complications that have occurred in patients with FE have been in about 40 % of the cases pin tract infections, 33 % nonunion, 11 % reactivation of the infection process, whereas in the group treated with intramedullary nail the only complication (15 %) was the reactivation of the infection process, for which reason it was necessary a new surgical procedure.
**Discussion** Our study has also confirmed better results for arthrodesis performed with intramedullary nail especially in terms of pain, function, and residual dysmetria rate.
**Conclusions** In conclusion arthrodesis, as described in literature, is confirmed as valid and effective surgery in difficult cases after periprosthetic infection of the knee.

### Computer-assisted navigation in orthopaedic oncology: preliminary experience

R. Piana*[1], S. Marone[1], A. Bruno[2], U. Albertini[1], E. Boux[1], M. Boffano[1], E. Brach Del Prever[1], G. Gino[1]

[1]Gruppo Piemontese Sarcomi, S.C. Chirurgia Oncologica e Ricostruttiva, AO CTO/Maria Adelaide (Turin, IT);
[2]S.C. Chirurgia Vertebrale, AO CTO/Maria Adelaide (Turin, IT)

**Introduction** Computer Assisted Orthopaedic Surgery (CAOS) has already been widely experienced in arthroplasties, spinal surgery and traumatology. Preliminary experiences have been presented in the surgery of pelvic and spinal tumours. The aim of this study was to evaluate the efficacy in terms of surgical margins adequacy, blood loss and length of surgery.
**Materials and methods** A central G2 chondrosarcoma of D5 vertebral body involving 3 posterior arches, a chordoma of the sacrum, a giant cell tumour of the pelvis, and an intrasacral epidermoid cyst were surgically treated at the Oncologic and Reconstructive Orthopaedics Department (CTO/Maria Adelaide Hospital, Torino Italy). All the tumours have been previously studied with CT and MR with contrast medium and have been resected 'en-bloc' with wide margins using CAOS navigation system Brainlab VectorVision Compact® (software VVSpine 5.5.1). The "CT-Fluoro matching" and the "paired point matching" systems have also been used. In the patient with D5 chondrosarcoma evoked potentials have been monitored during the surgical resection of the intermediate pedicles and the 3 posterior arches. Complications, surgical margins, length of surgery, blood loss were evaluated.
**Results** No major complications were described, resection levels were easily identified (although the initial learning curve), resection margins were judged corrected and adequate both macroscopically and microscopically by the pathologist. The average length of surgery and blood loss seemed to be lower, compared to similar cases treated in the traditional way in our experience.
**Discussion** These preliminary results confirm the importance and usefulness of CAOS in orthopaedic oncology, mostly in the resection of anatomically challenging tumours. Variables to be considered are: histotype, compartment, dimensions of the tumour, response to adjuvant and neo-adjuvant treatments, and surgeon's experience.
**Conclusions** More cases, eventually in a multicentric study, are mandatory to definitively affirm if this technique can also decrease local recurrence rate and improve overall survival.

### Modular prosthesis with a silver coating for periarticular reconstruction in septic prosthetic and post-traumatic failures

N. Mondanelli*, D. Campanacci, G. Caff, G. Beltrami, G. Scoccianti, R. Capanna

Ortopedia Oncologica e Chirurgia Ricostruttiva, Ospedale Careggi, CTO (Florence, IT)

**Introduction** Infection in orthopaedic surgery is a dreadful complication. Patients are often subjected to several surgeries with prolonged antibiotic treatment, and the risk of persistent infection and debilitating functional outcome is high. Often, a massive bone defect coexists, linked to the need to perform extensive debridement to remove necrotic or infected bone. The antimicrobial activity of silver ion has been known since ancient times (silver vases and cisterns for drinking water) and in recent years has been revived in everyday life (toothbrushes, underwear) as well as in medicine (wound dressings).
**Materials and methods** Recently, we developed an evolution of the modular prosthesis MegasystemC (Waldemar Link, Hamburg, Germany) with a silver coating (PorAg) and, at our Centre, from June 2010 to February 2012 were operated on 10 patients with septic arthroplasty (4 cases, 2 hips and 2 knees) or septic meta-epiphyseal post-traumatic deformity or nonunion (5 cases, 1 proximal and 4 distal femur) and on a patient affected by an epithelioid hemangioendothelioma of the distal femur with a previous infected biopsy. One patient with subtrochanteric nonunion was subjected to only 1 surgery before resection and modular silver-coating prosthesis, while in all other cases the number of previous surgeries ranged from 3 to 8. In 7 cases the infection had resolved, while in 3 cases the infection was persistent (1 knee arthrodesis prosthesis as a results of septic knee megaprosthesis and the subtrochanteric nonunion) and it was decided to revise them one-stage.
**Results** In 8 cases the reconstruction was performed with an articulating prosthetic joint (3 proximal femur and 5 knee megaprosthesis of the distal femur) and in 2 cases with a knee arthrodesis prosthesis. Monitoring of inflammatory markers (ESR, C-reactive protein, fibrinogen) showed resolution of the infection in all cases.
**Discussion** From the clinical point of view, all patients were satisfied with surgery. Radiographically, there are no signs of loosening or periprosthetic bone resorption.
**Conclusions** the preliminary results of such a limited group of patients are encouraging and demonstrate that the use of silver coating prosthesis may be indicated in the reconstructions of periarticular loss of substance in septic failures, making single-stage revision surgery safer.

### *C29—KNEE 5*

### “Over the top” reconstruction of the ACL with single or doubled hamstrings: 12-year follow-up

A. Ruffilli*, R. Buda, M. Cavallo, D. Luciani, F. Tesei, M. Baldassarri, F. Castagnini, S. Giannini

II Clinica, Istituto Ortopedico Rizzoli (Bologna, IT)

**Introduction** ACL reconstruction techniques increased and have deeply been modified during the last years. Nevertheless the points to take into account are the same: preserving articular stability and physiological articular biomechanics to avoid osteoarthritis by meniscus and cartilage damages. The aim of this study was to evaluate clinical and radiographic scores of single or doubled semitendinous and gracilis autograft ACL reconstruction after 12 year follow-up.
**Materials and methods** 51 patients were enrolled and treated by non anatomical ACL reconstruction with the “over the top” technique. Mean age was 29.2 years. All the patients were similar for age, kind of lesion, time between trauma and surgery. 20 patients (group 1) underwent single ST and GR ACL reconstruction, and 31 cases (group 2) underwent the same procedure with o doubled hamstrings. Patients were evaluated pre-operatively and periodically until the maximum follow-up (12.1 ± 1.6 years). Clinical evaluation was performed according IKDC, KOOS, Tegner score and KT-1000 arthrometer. Radiographic evaluation using IKDC score was performed at the maximum follow-up.
**Results** At the final follow-up, subjective IKDC was 76.6 ± 21.9 in the first group and 88.9 ± 10.0 in the second group ($p = 0.009$). KOOS score was 82.6 ± 18.7 in the first group and 92.4 ± 9.2 in the second group ($p = 0.0016$). Objective IKDC score showed higher percentage of normal knee in the second group. The second group highlighted a better rotational stability at the follow-up, as Pivot shift test established. Radiographic evaluation showed lower incidence of degenerative disease in the second group, both in the medial compartment ($p = 0.01$) and the lateral one ($p = 0.037$).
**Discussion** OTT technique, although not anatomical, showed to be good in controlling antero-posterior and rotational translation of the knee. The single OTT technique showed a poor ability in restore a sufficient stability, especially the rotational aspect, with higher risks of long-term arthritic degeneration.
**Conclusions** Doubled hamstrings ACL reconstruction showed better subjective and objective results and lower incidence of long-term arthritic degeneration. Biomechanical peculiarities of hamstrings tendons and OTT femoral tunnel require a doubled reconstruction to guarantee rotational stability and to lower the incidence of long-term degenerative disease.

### Treatment of osteochondritis dissecans of the knee with arthroscopic bone marrow-derived cells transplantation (“One step” technique): results and T2 mapping evaluation

R. Buda*[1], F. Vannini[1], M. Cavallo[1], F. Tesei[1], M. Battaglia[2], A. Olivieri[1], S. Giannini[1]

[1]II Clinica Ortopedica e Traumatologica, Ospedale Ortopedico Rizzoli (Bologna, IT);
[2]Servizio di Ecografia, Radiologia e Radiodiagnostica, Istituto Ortopedico Rizzoli (Bologna, IT)

**Introduction** Osteochondritis dissecans of the knee (OCD) is an acquired osteochondral pathology of a femoral condyle. The aetiopathogenesis and the ideal management of this pathology remain uncertain. During the juvenile age non surgical treatment could be adequate, nevertheless surgery is often required especially in adults. Although satisfactory results, the treatments traditionally used, such as mosaicoplasty or Autologous Chondrocyte Implantation still present disadvantages difficult to overcome and results inferior to those obtained by the same procedures in the treatment of traumatic osteochondral lesions. Possibly this data is related to vascular disorders at the base of OCD. Aim of the study was to correlate the clinical and RM-T2 mapping results of a group of patients treated by bone marrow-derived cells transplantation (BMDCT).
**Materials and methods** 9 patients (25.4 ± 12.6 years) affected by OCD of the medial femoral condyle, were treated between 2006 and 2009 by BMDCT. The bone marrow-derived cells were harvested from posterior iliac crest, concentrated in the operatory room, loaded on a hyaluronic acid scaffold with platelet-gel and arthroscopically implanted in the same surgical session. Patients evaluation included IKDC, KOOS score, X-rays and MRI (Mocart score) preoperatively and at 37.3 ± 16.9 months of follow-up. At the final follow-up qualitative evaluation by RM-T2 mapping was also performed.
**Results** IKDC score improved from 47.8 ± 10.0 to 76.8 ± 15.7 ($p = 0.0005$), while KOOS from 58.5 ± 12.9 to 82.1 ± 17.9 at follow-up ($p = 0.0005$). MRI (Mocart score) showed a satisfactory regeneration of the lesion site, while T2 mapping confirmed the presence of hyaline cartilage at the reparative site.

**Discussion** Treatment of osteochondritis dissecans of the knee with arthroscopic BMCDT (“One step” technique) shows satisfying results under clinical and imaging aspects. RM-T2 mapping showed the regenerated hyaline cartilage on the articular surface and an underlying filling of the subchondral zone with newly bone regeneration.
**Conclusions** The “One step” technique demonstrated to be a good and reliable option for the treatment of OCD, furthermore it overcomes many disadvantages of the techniques traditionally used. A larger case series and long term evaluation are needed in order to confirm the validity of the procedure.

### Treatment of large knee osteochondral lesions with a new biomimetic scaffold: minimum 2-year follow-up multicenter study

E. Scaravella*[1], M. Delcogliano[1], C. De Biase[1], D. Marotta[2], P. Marenghi[1], A. Delcogliano[3]

[1]Azienda Ospedaliero Universitaria di Parma (Parma, IT);
[2]Ospedale San Carlo, IDI (Rome, IT);
[3]Casa di Cura Guarnirei, Ospedale San Carlo, IDI (Rome, IT)

**Introduction** Large osteochondral articular defects are difficult to treat and present a high rate of complications, such as post-traumatic joint congruity, axial misalignment and possibly arthritis. In the last 20 years many different approaches have been proposed to restore the cartilage lesions, and satisfactory results have been obtained. However, the treatment of osteo-cartilaginous lesions is even more problematic, because tissue damage is extended also to the subchondral bone, involving two different tissues characterized by different intrinsic healing capacity. The objective of this clinical study was to test safety and performance of a newly developed type-I collagen-hydroxyapatite (HA) nanostructural bio-mimetic osteochondral (O.C.) scaffold which reproduces cartilage-subchondral bone morphology.
**Materials and methods** A gradient composite O.C. scaffold, based on type-I collagen-HA, was obtained by nucleating collagen fibrils with hydroxyapatite nanoparticles at physiological conditions. 23 cases with large knee osteochondral lesions were treated as salvage procedure with scaffold implantation. The lesions size went from 4 to 8 $cm^2$. All the patients achieved minimum 2-year follow-up and were clinically evaluated using the International Repair Cartilage Society score.
**Results** IKDC objective score improved after 2 years showing a normal or nearly normal knee in 80 % of patients. Similar results were obtained with the IKDC subjective score MRI evaluation demonstrated good bone and cartilage formation and only in the failed case no integration of the graft was found.
**Conclusions** This open one-step surgery was used for the treatment of large osteochondral defects. The results of this technique at short follow-up are very encouraging and show satisfactory results even in massive ostechondral defects.

### Lateral unicompartmental knee arthroplasty: indications, surgical technique and short/mid-term results

C. Tsatsis*[1], G. Gazzotti[1], G. Matino[1], V. Digennaro[1], G. Sacchetti[2], F. Catani[1]

[1]Policlinico (Modena, IT);
[2](Bologna, IT)

**Introduction** Medial Unicompartmental Knee Arthroplasty is increasingly used for medial osteoarthritis in varus knee and in medial unicondilar osteonecrosis, with excellent or good results at mid/long-term follow-up. Lateral Unicompartmental Arthroplasty (LUA) is much less used (2.5–6 %), for the lower incidence of isolated lateral osteoarthritis and for its technical features of implant as well. In this study we report our indications, the surgical technique and the short and mid-term results.
**Materials and methods** At the Orthopaedic Department of University of Modena, from January 2002 to September 2011, in a total of 560 Unicompartimental Knee Arthroplasties, 20 patients were operated with LUA (3.5 %). We have used a lateral parapatellar approach; all components were cemented. Our indications were: valgus deformity < 15° (passively correctable), lateral condropaty of III°-IV° according to Outerbridge classification, with a radiologically satisfactory medial and patello-femoral joint space. The average age was 59 years old (range 41–82), of wich12 males and 8 females; the mean BMI was 26.7 (range 21–34). Also, in 3 of our patients there was a post-traumatic gonarthrosis.
**Results** We re-evaluated 17 patients for a mean follow-up of 37 months (range 6 months–9 years). Clinical and radiographic assessment was performed. All patients were evaluated according to the Knee Society and Oxford Knee Scores. We obtained excellent-good results in 82.4 % of cases (mean KSS 82.8/mean ONS 39.4), the mean VAS was improved from 8 pre-operatively to 2.1 post-operatively. Mean valgus deformity was 7° (10° pre-operatively) and mean posterior sloope was 3° (5° pre-operatively). No revisions were performed. We observed one case of joint stiffness and two cases of painful knee.
**Discussion** LUA is a much rarer surgical treatment than the medial one, used in carefully selected patients. Based on our observations and the literature as well, there are not significantly different results of LUA compared to Medial Unicompartmental Knee Arthroplasty.
**Conclusions** LUA is a valid treatment for lateral knee osteoarthritis, accompanied early resolution of pain and early knee mobilization, but it’s necessary to acknowledge and respect some important technique details. According to our experience LUA represents a reliable and successful option, compared to the other available procedures (arthroscopic debridement, osteotomy, total knee arthroplasty).

### Tantalum uncemented tibial component in total knee arthroplasty: mid-term results

D. Lup*[1], P. Caldora[1], R. Guarracino[1], E. Bartoleschi[2], G. Prestia[3]

[1]AUSL 8 Arezzo (Arezzo, IT);
[2]U.O. Ortopedia e Traumatologia, Policlinico Le Scotte, Università degli Studi di Siena (Siena, IT);
[3]U.O. Ortopedia e Traumatologia S.U.N. (Naples, IT)

**Introduction** The porous tantalum is a very important alternative in prosthetic surgery of the knee. The purpose of this prospective study was to evaluate the clinical and radiographic results in the short and in the medium term of tantalum uncemented tibial component in total knee arthroplasty.
**Materials and methods** In our Unit, from July 2005 to July 2011, 98 total knee arthroplasty were performed with the Nexgen LPS system (Zimmer, Warsaw, Indiana, USA) using the tibial component in cementless tantalum Trabecular Metal™ with polyethylene monoblock. We have always given intra-operative indication in the use of this tibial component according to the quality of the patient’s bone. In all cases the polyethylene was PS. Forty patients were female and 57 were male and their average age was 61 years (range 46–75). The average follow-up was 40 months (range 6–78). The protocol provided a clinical and radiographic evaluation preoperative after

3 months and annually, and only clinical evaluation after 1 and 6 months post-operatively. The clinical and radiographic evaluation pre-and post-surgery was performed using the Knee Society Scores.
**Results** We didn't find any intra-operative complications. We performed 1 revision (1 %) of the component in TM due to late infection. The average score of the Knee Score increased from 40 points (28–60) pre-op. to 92 points (58–100) at last follow-up. In 97 % of the cases we obtained excellent and good results. At radiographic evaluation we didn't observe any case of periprosthetic radiolucent line greater than 1 mm, periprosthetic osteolysis and aseptic loosening of the implants.
**Discussion** The TM is a material that has a high coefficient of friction and mechanical strength, 80 % porosity, and modulus of elasticity similar to bone; these features allow the immediate primary stability and a lasting osseointegration. Furthermore, the particular type of manufacture of this tibial component, which is a monoblock with polyethylene, is able to minimize the friction and therefore limit wear that occurs at the interface polyethylene-metal back. These features make the tantalum tibial component a valid alternative to the cemented components in selected cases.
**Conclusions** The good clinical and radiographic results obtained, lead us to believe that the use of tantalum tibial component may be a good solution, especially for the young patient suffering from knee arthrosis, but we need longer follow-up to evaluate the percentages of long-term failure.

### *C30—PAINFUL PROSTHESES 3*

### The effect of drainage on postoperative pain and bleeding of knee replacements

G.L. Canata*, V. Casale, A. Chiey

Centro Traumatologia Sport Koelliker (Turin, IT)

**Introduction** In total knee replacement surgery the use of postoperative drainage is common. The efficacy of this type of procedure on postoperative pain and bleeding is still debated.
**Materials and methods** 62 patients, mean age 70 years (range, 40–83), 19 males and 43 females operated between 2007 and 2009 were randomized in two groups: in Group A (8 males and 23 females, postoperative drainage was inserted; in Group B (11 males and 20 females) no drainage was inserted. In all patients, a non-cemented NexGen Zimmer total knee arthroplasty was implanted. A subjective pre- and postoperative functional evaluation using the Knee Injury and Osteoarthritis Outcome Score (KOOS) was carried out, with a mean follow up of 30 months; Furthermore, reductions in haemoglobin were recorded in every patient between the preoperative and the first postoperative week. A prospective randomized study was performed. *t* test was used for statistical analysis.
**Results** The mean KOOS postoperative score was 84 (St Dev 16) in Group A with drainage and 86 in Group B without drainage (St Dev 20). In Group A, the hemoglobin underwent a reduction of 4.2 g/dl; in Group B the reduction was 3.2 g/dl.
**Discussion** Not applying drainage in the immediate postoperative time did not determine an increase in complications. No significant variations in blood loss were observed. The KOOS evaluation did not show significant differences.
**Conclusions** The lack of drainage after knee replacement surgery does not determine significant differences in postoperative results.

### I-ONE therapy in patients undergoing total knee arthroplasty: results at 12 months

V. Pesce[1], A. Notarnicola*[1], S. Setti[2], L. Moretti[1], G. Vicenti[1], B. Moretti[1]

[1]Dipartimento di Neuroscienze e Organi di Senso, Cliniche Ortopediche del Policlinico Universitario di Bari, Università degli Studi "Aldo Moro" di Bari (Bari, IT);
[2]Laboratorio di Biofisica Clinica, ICEmB-Igea (Carpi, Modena, IT)

**Introduction** The aim of this study was to evaluate if the application of biophysical stimulation is helpful in enhancing the standard rehabilitation in patients undergoing TKA, leading to relief of pain and a complete return to daily activities.
**Materials and methods** A prospective, randomized, controlled study on 30 patients undergoing TKA was conducted. Patients were randomized in experimental (I-ONE therapy) or control group. All the patients received the standard rehabilitation program. Treatment outcome was assessed using the Knee Score, Functional Score, SF-36 and VAS before and at 1, 2, 6 and 12 months after surgery. Joint swelling and drug assumption were evaluated. The descriptive analysis of variables was performed by calculating mean value and standard deviation; comparison between the two groups was made by two-tail heteroscedastic student's *t* test. Analysis of variations for each individual subject with respect to baseline was performed and compared between groups by two-tail Student's *t* test. A *p* value of 0.05 was considered statistically significant.
**Results** Pre-operatively, no difference was observed between groups for age, sex, weight, height, KNEE score, VAS, SF-36 and joint swelling. The Knee Score shows significantly positive outcomes for the I-ONE group at 2 and 6 months; VAS and SF-36 score at all follow-up. In the I-ONE group drug use was less frequent and joint swelling resolution was faster. The positive results are maintained also in the long term.
**Discussion** Total knee arthroplasty (TKA) is often associated with a severe inflammatory reaction which, unless controlled, leads to persistent pain up to a year after surgery. Standard and accelerated rehabilitation protocols are currently being implemented after TKA, but no consensus exists regarding the long-term effects. Biophysical stimulation with pulsed electromagnetic fields has been shown to exert an anti-inflammatory effect, to promote an early functional recovery and to have a positive long-term effect in patients undergoing joint arthroscopy.
**Conclusions** The results of the study show early functional recovery in the I-ONE group. I-ONE therapy should always be considered after TKA to limit joint inflammatory reaction, to resolve pain, to shorten the recovery time and, ultimately, for joint preservation.

### *C31—SHOULDER AND ELBOW 7*

### Elbow arthrolisis: our experience in 20 cases

M. Zanlungo*, E. Finardi, F. Nasi, F. Caracciolo

Istituto di Cura Città di Pavia (Pavia, IT)

**Introduction** Surgical operation program require an attentive evaluation and complete X-rays study, 3 dimensional ACT, MNR and if it is symptomatic EN/EMG of ulnar nerve.
**Materials and methods** From January 2003 to December 2011 we operated 20 pts: 18 were 72 years old, 12 men, 8 women. We had 12 dislocation outcomes (2 inveterated 8 with ectopic calcifications 8

fractures outcome with whole humeral troclea. We used 2 columns Morrey's technique. The release of ulnar nerve was followed by a complete anterior capsulotomy to the epicondiloideals muscles insertion. Coronoid fossa must be cleared from all fibrous tissues and possible calcifications must be removed. If the extension is limited the posterior fossa must be cleared. In fractures outcomes, we prosthesized only the humeral condyle in 2 cases; in one 18-year old boy we remolded humeral condyle and removed excess of bone callus. In 3 cases radial capitulus was resected. Physiotherapy treatment began immediately post-operatively and was completed by the use of a Quenquel type dynamic tutor.
**Results** 18 cases recovered functional joint range; 2 bad results were caused by calcifications relapse. We must still review all cases of this study.
**Discussion** Surgical arthrolisis of the elbow requests a good collaboration of the patient who must be informed about the possibility of an incomplete result or the relapse of stiffness. The ulnar nerve release is an unavoidable choice also in case of arthroscopic surgery.
**Conclusions** The fractures-dislocations outcomes can be treated if, besides arthrolisis, the reconstruction of congruent and steady joint is predictable. The prosthetic reconstruction of the humeral condyle, in addition to the capitulum, is actually possible. We do not have experience on humeral troclea reconstruction.

### Localized osteoporosis due to reduced functionality of upper limb in women over 60 affected by rotator cuff disease or scapular-humeral arthrosis

V. Bottai*, S. Giannotti, G. Dell'osso, G. De Paola, E. Pini, G. Bugelli, G. Guido

Ortopedia e Traumatologia, II Università di Pisa (Pisa, IT); Clinica Ortopedica, Università di Pisa (Pisa, IT)

**Introduction** In scientific literature, non-use osteoporosis due to reduction of loading forces is mainly reported in patients with serious neurological injuries, in particular at lower limbs level. Subsequently, in case of decreased functionality, any skeletal segment can be reabsorbed, even if to a minor extend compared to the neurological patients cases.
**Materials and methods** We analyzed two groups of patients. In both cases, patients were women over 60. The first group was of women with rotator cuff injury or scapular-humeral arthrosis with a presurgical constant score below 50. The second group, instead, was of patients over 60 with hip and knee arthrosis disease with constant score of left or right shoulder above 70. All patients were submitted to clinical evaluation, humeral and femoral Cortical Index measurement and femoral, column and radius DEXA scan.
**Results** The first group values of both humeral Cortical Index and radius DEXA scan were lower than the second group ones—patients with preserved shoulder function—showing that the bone mass reduction is related to reduced use. Radius DEXA values were also lower compared to those of column and femur in the same patients, proving that it is a localized bone resorption.
**Discussion** The proximal third of the humeral fractures are, in osteoporotic patients, constantly increasing and involve patients relatively younger than the ones affected by fragility fractures of other body parts (i.e. : femur neck). Most of those injuries don't need surgical treatment. So far, there isn't a general common treatment choice for surgical injuries. There is instead a general common acknowledgement that the bone mass alteration is one of the main predictive factors of a bad surgical result. In spite of this, until today there is no evaluation method to measure the mineral bone density of the proximal third humerus.
**Conclusions** If we take into consideration that the result of the relation between the values of radius DEXA scan and humeral Cortical Index in patients with good use of the upper limb is reliable, it is possible to make a prevision of humeral BMD, using, as a reference, the value of humeral Cortical Index of the limb affected by articular and peri-articular scapular-humeral disease.

### Mid-distal humeral shaft fractures: reduction and synthesis with two parallel angular stability plates through posterior approach

G. Campochiaro*, G. Gazzotti, G. Matino, C. Rovesta, F. Catani

(Modena, IT)

**Introduction** We treated mid-distal humeral shaft fractures with reduction and internal fixation with two parallel angular stability plates through posterior approach. The purpose of this study was to evaluate our results by comparing it with other techniques used.
**Materials and methods** We reviewed 18 patients (age 14–81) with mid-distal humeral shaft fracture type A-B according to AO classification. These fractures were treated surgically from September 2006 to December 2011. Ten cases were treated with 2 parallel plates implanted trough a posterior surgical approach. 8 cases were treated with screws and a single lateral plate positioned trough lateral surgical approach. Functional outcomes were assessed with DASH questionnaire, MAYO score, elbow ROM and stability, strength compared to controlateral side and by reviewing the radiographs.
**Results** We found non-significant statistically differences between the two groups of patients. We obtained good clinical results, with low number of complications. The recovery of elbow ROM was complete in most of the patients. The average DASH questionnaire was 14, in the group treated with two parallel plates, and 17, in the group treated with lateral plating. Three cases of radial nerve compression were noticed in the first group, one case in the second one. In all cases the recovery was complete.
**Conclusions** Both techniques showed good clinical results. The treatment with two parallel angular stability plates represents a valid procedure. It ensures greater stability of the sinthesis and allows an early mobilization of the elbow. However it requires a more complex surgical approach and longer surgical time.

### The treatment of fractures of the proximal humerus with Philos plates: clinical and radiographic results at a distance

M. Rendine*, R. Di Virgilio, E. Coppari, G. Condarelli

(Rome, IT)

**Introduction** Displaced fractures of the proximal humerus, especially if associated with dislocation of the glenohumeral joint, offering the orthopaedic trauma surgeon-major difficulties in choosing the most appropriate treatment. The choice between different methods of internal fixation or prosthetic replacement is still a matter of debate among trauma surgeons from around the world.
**Materials and methods** The authors propose a review of recent literature and their clinical and radiographic results at a distance in cases of fractures of the proximal humerus treated surgically with a plate with angular stability Philos. The authors report their series, which since 2007 has had more than 70 cases was average of

3.5 years. Only cases with 3 and 4 fracture fragments were considered. The mean age of patients was 63 years (min. 35–max. 82). We have considered all the possible complications, ranging from those related to neurovascular and implant failure.

**Results** All patients were followed in time with X-ray controls and clinical assessment by the Constant score and the Simple Shoulder test. In assessing the results we found that '87 % of patients reported excellent or good, 13 % unsatisfactory results. 25 % of patients developed avascular necrosis of the humeral head but 80 % of these patients reported a value of the Constant score from good to excellent.

**Discussion** Locking plate fixation is a good surgical option for the management of proximal humerus fractures. Careful adherence to technical aspects because the use of better bone stock in the inferomedial region of the humeral head may reduce hardware complications, especially in patients with osteoporosis.

**Conclusions** The authors conclude that the fixation with plates with angular stability is a surgical solution is also valid for older patients with fractures in three four fragments, whereas the clinical results of shoulder replacement are not yet so brilliant. Several precautions in the placement of screws and plates also allow you to avoid some complications such as perforation of the humeral head by the screws.

### Dislocation of ulnar nerve at the elbow: a new surgical technique

N. Galvano*, A. Parlato, M. Butera, A. D'arienzo

Clinica Ortopedica, Università Palermo (Palermo, IT)

**Introduction** The dislocation of ulnar nerve at the elbow is responsible of a neurological symptomatology similar to that of the classical compression of the nerve, also it is lighter than that and with so vanished aspects to be made difficult its diagnosis. The purpose of the study was to propose a new surgical technique.

**Materials and methods** The diagnosis is based on the clinical examination characterized by the verification with the pathology of the partial or complete spillage of the nerve from his lodging during the movements of bending of the elbow. The essential examination for the diagnosis is a dynamic echography. We used this technique in 12 cases of post traumatic dislocation, from 2007 to 2010. All cases were examined with VAS and a scale of functional recovery at 6 months and 1 year after treatment.

**Results** The proposed surgical treatment is the liberation of the ulnar nerve and its reinsertion in the anatomical center through the use of a big fibrous-adipose edge that is sutured with disconnected points reconstructing so the epitrochlear-olecranon ligament. We used this technique in 12 young patients with very satisfactory results.

**Discussion** The anterior transposition deep is the surgical treatment of routine mostly used and recommended but the treatment with liberation and reinsertion in the anatomical center of the nerve in young patients gives satisfactory results because it is more conservative and it clearly engraves in smaller way on the post-operating muscular strength.

**Conclusions** The plastic of the epitrochlear-olecranon ligament through the use of a big fibrous-adipose edge is the technique more anatomic that allows an optimal functional recovery of the elbow.

### Endoprosthesis in radial head fractures

R. Valentini*, G. Fancellu, S. Bernobi, P. Dordolin

Clinica Ortopedica e Traumatologica, Università degli Studi di Trieste (Trieste, IT)

**Introduction** The radial head works together with other bones, ligaments and tendons that are located around the elbow to stabilize the joint. The indication surgical osteosynthesis is traditionally a shift greater than 2 mm and when more than 30 % of the area is concerned, while in the fracture Mason 3 type, separated and comminuted with more than three fragments is indicated resection or prosthetic replacement with endoprosthesis.

**Materials and methods** In the past years we have used silastic implants and alumina endoprosthesis in over a hundred cases. Over the past 6 years we have treated 15 cases of isolated fracture of the radial head and 12 cases associated to other fractures or dislocation (coronoid, olecranon, coronoid and olecranon, distal end of humerus) with metallic non-modular endoprostheses in 18 cases and with modular prosthesis in 9 other cases.

**Results** The results were evaluated according to the Morrey score. 16 patients resulted to be excellent, 5 patients were good, 3 were sufficient and 3 bad enough. Complications met were one removal of the endoprosthesis for stiffness and three heterotopic ossifications.

**Discussion** We prefer to perform an articular replacement rather than resection of the capital to prevent the shortening of the radius, subluxation of the distal radio-ulnar joint and instability of the elbow.

**Conclusions** The substitution of the resected radial head with endoprosthesis provides optimal stable results over time in non-associated fractures. Always evaluate the presence of lesions associated with CT or MRI. Perform always intraoperative test of ligament stability. If it is an apparent MCL injury the synthesis of the fracture or the endoprosthesis replacement prevents revision of the MCL. Always have to reconstruct the coronoid. The prosthetic replacement of the radial head fracture allows rapid mobilization and fast recovery.

### Post-traumatic stiffness in radial head fractures and fracture-dislocations of the elbow

S. Barreca, L. D'andrea, L. Miccihé, F.R. Campo, G. Orlando, C. Casablanca, M.A. Rosa*

Scuola di Specializzazione in Ortopedia e Traumatologia, Sezione di Ortopedia e Traumatologia, Dipartimento Specialità Chirurgiche, Università degli Studi di Messina (Messina, IT)

**Introduction** Post-traumatic stiffness is always a redoubtable issue with radial head fractures and radial head fractures with dislocation of the elbow. Prevention of stiffness is not only based on surgery but also on medical therapy and rehabilitation after surgery.

**Materials and methods** The authors refer to a series of 10 patients with isolated fracture of the radial head in 8 cases and fracture-dislocation of the elbow in 2 cases. Fractures and fracture-dislocations were classified according to Mason: 3 type I, 3 type II, 2 type III and 2 type IV. In 4 cases, observed immediately after the trauma, medical treatment was performed with Indomethacin 75 mg/day orally in three doses for 7 days, in 6 cases, observed by the authors after 6 months from the trauma and treated by other surgeons, the Indomethacin was used at the time of the second operation. Surgical treatment used a screw osteosynthesis in 2 cases and a total and/or subtotal radial head removal in 8 cases. Active rehabilitation was started in all cases immediately after surgery.

**Results** Results were evaluated with radiographic and clinical examinations performed at 1, 6 and 12 months after treatment. Valgus deviations with extended elbow and the limitations of pronation and supination were considered. The function of the elbow and the pain during active movements were assessed according to the Mayo Elbow Performance Score.

**Discussion** The choice of treatment depends on several factors: age of patient, general conditions, type of fracture. The therapeutic target must be in keeping with the anatomy and biomechanics of the elbow. Safeguarding the radial head is indicated in fractures of Mason type I and II, while in fractures of Mason type III and IV, the comminution of the fragments and the dislocation require the intervention of radial head removal within 24-48 h after trauma to prevent hetero topic calcifications and a secondary stiffness. Rehabilitation should be active and actively assisted with prolonged post-surgical pain therapy.
**Conclusions** Osteosynthesis with the use of screws prevents a valgus deviation that may be a constant factor after radial head removal. According to the authors, early rehabilitation and therapy with Indomethacin are the basis for the prevention of stiffness resulting from fractures of the radial head with or without dislocation.

### *C32—HAND AND WRIST 1*

### Nonunion of the scaphoid: treatment approach

N. Galvano*, S. Moscadini, A. Parlato

Clinica Ortopedica (Palermo, IT)

**Introduction** Authors highlight the experience of over 60 cases of scaphoid nonunion treated surgically. They emphasize the importance of adequate and early diagnosis through the use of MRI, as well as x-ray examination and the importance of correct classification, preferring Herbert classification.
**Materials and methods** We treated from November 2007 to December 2010, 60 nonunions of the carpal scaphoid always using the closed technique with percutaneous synthesis and the use of PRP after milling of the body. We obtained the compaction of the fracture through the screw.
**Results** The check has been performed at 6 months and at 1 year, and it showed the healing of the nonunion in 96 % of cases. Only in 4 % there was a failure due to the position of the fracture site (proximal polo).
**Discussion** Today, in our experience the mere synthesis in nonunion treatment is no longer acceptable due to enormous failures. Other traditional techniques, still in use, resulted in a cure, although with large functional limitations. The use of PRP, certainly determines a bone healing thanks to an osteogenic stimulation.
**Conclusions** Up to now, the percutaneous use of a screw with bone graft of PRP is an extremely valid option for the healing of pseudoarthrosis of the scaphoid, although in skilled hands.

### Developments in surgical treatment of distal radius fractures using volar plates and screws: our 7-year experience

G. Bruno*, A. Tomarchio, D. Ghargozloo, E. Marchese, L. Cannavò

Sezione di Ortopedia e Traumatologia, Dipartimento delle Specialità Medico Chirurgiche, Azienda Ospedaliera Universitaria Policlinico Vittorio Emanuele (Catania, IT)

**Introduction** The management of distal radius fractures is changed significantly in recent years: the conservative treatment, frequently used in the past, has shown poor results in a considerable number of patients. Therefore, especially in young patients the surgical approach has become more frequent. Furthermore, the spread of new methods of osteosynthesis and of modern materials have greatly improved the ORIF technique. This is now the gold standard compared to other techniques such as external fixation, associated or not to percutaneous fixation.
**Materials and methods** In our department, from January 2005 to January 2012, a total of 195 distal radius fracture patients were treated with ORIF technique using a volar approach (87 % type B and C according AO classification system): 132 male and 63 female with mean age 41 years (17–71). The patients were reviewed with follow-up between 9 and 84 months, outcomes were evaluated using clinical evaluation, DASH and Modified Mayo wrist score, radiographic assessment at follow up. In 28 cases was performed CT scan pre-op and after about 12 months post surgery.
**Results** Of the 195 patients 160 have been evaluated after the surgery; with assessing functional and radiological follow up was documented complete healing, with excellent/good result, in 94 % of patients. Rare cases were unsatisfactory: 9 patients experienced slight limitation of pronation/supination, transient irritation of median nerve was observed in 3 patients, there was evidence of early osteoarthritis in 5 patients. These complications occurred in patients with fractures C2 and C3 type, associated with other bone fracture and/or soft tissue injury. We removed the plate in 5 patients at their request.
**Discussion** The aim of our study was to evaluate the functional outcomes of distal radius fractures treated with angle-stable volar plate. In these years we used various types of plates, thanks to scientific and technological innovations. These improvements allowed better design and low profile plates, as well as the possibility to fix the screws to the plate with variable angle.
**Conclusions** Angle-stable volar plate is an excellent system to treat the distal radius fractures: is possible to obtain the anatomical reduction, reposition the dislocated fragments and to have an early functional recovery. The plates that we used over the years showed different characteristics. The final result is certainly conditioned by both the type of fracture and the surgical technique.

### Rizoarthrosis: our experience with 104 patients treated with suspension arthroplasty by Weilby's technique

G. Anelati, A. Caffi, T. Kapotsis, P. Rota, L. Valle*

AO Bologni Seriate, Ospedale M.O.A. Locatelli (Piario, Bergamo, IT)

**Introduction** Trapezio-metacarpal arthritis, rizoarthrosis, is a frequent and invalidating disease and represents about 10 % of all arthritis localizations. From literature one can infer that there are several treatment possibilities for this disease: arthrodesis, arthroplasty, tendon interposition suspension arthroplasty, trapeziectomy; none of these techniques proves to be superior to others in terms of results, with a follow up longer than 1 year, or in terms of complications. Our experience is based on 104 patients suffering from rizoarthrosis treated with Weilby interposition tendoplasty; we could not find any reported study in literature with such a broad survey and a median follow-up so long.
**Materials and methods** We treated 104 patients suffering from rizoarthrosis treated with Weilby interposition tendoplasty from 1996 to 2011. All patients were followed up for a minimum of 1 year using the DASH questionnaire, radiological evaluation and a specific patient satisfaction questionnaire. We further evaluated objectively articularity and grip strength.
**Results** Patient satisfaction, articulation recovery and grip strength after at least 1 year reached high values.
**Discussion** Our study demonstrates that tendon interposition arthroplasty as described by Weilby turns out to be a valid alternative to treat primary thumb carpometacarpal osteoarthritis considering low incidence of complications, patient satisfaction, articular congruence between bases of 1st and 2nd metacarpal bones as evidenced by long-term radiological examination, and considering the possibility to treat also carpal arthritis which involves also scaphoide, trapezoid and capitate.
**Conclusions** The extent of our cases study and the length of the follow-up allow considering tendon interposition arthroplasty, as described by Weilby, a valid treatment for trapezium metacarpal arthritis.

### The scapho-lunate arthrosis of the radius: selective arthrodesis with excision of the scaphoid

N. Galvano*, G. Caradonna, A. Parlato, G. Vercio, M. Lauria

Clinica Universitaria Palermo (Palermo, IT)

**Introduction** The degenerative alteration of the joint-radio-scapho-lunate is very common and can be related to several post-traumatic or degenerative diseases. Various techniques are proposed till the use of carpal implants. Our proposal provides a selective arthrodesis of the radio scafo with lunate excision of the distal pole of the scaphoid. In addition, our study provides a description of the different stages of the technique by presenting the results at a distance.
**Materials and methods** We have selected, from January 2007 to December 2010, 12 patients with an alteration of the radio-scafo of medio-lunate with normal carpal articulation. This condition was the result of traumatic events and degenerative pathologies. There was a second-degree DISI. All twelve patients were studied by X-rays in 2 pts, CT and MRI, with particular attention to the DISI is an arthrodesis is performed, due to the dorsal access, selective and stabilized with K wires and subsequent excision the distal pole of the scaphoid. The evaluation was performed at 6 and 12 months by VAS scale and the DASH.
**Results** In an evaluation at 12 months postoperatively 10 had no pain, pain during exercise, and 1 accused 1 during work activities. The average mobility of the wrist in flexion was 32° and 35° in extension. The muscle strength by 80 % compared to contralateral. In all patients consolidation of arthrodesis was obtained. Only after 24 months, in 2 patients a medio carpal arthrosis secondary to DISI was noted, already associated with a pre-existing one.
**Discussion** Few surgical techniques may be indicated in case of damage to scafo of the radio-lunate articulation with the spacing midcarpal healthy; between these arthrodesis and wrist prosthesis are currently the most used although with severe functional limitations.
**Conclusions** Arthrodesis radio scafo with lunate excision of the distal scaphoid is an excellent indication. Particular importance should be given to resection of the distal pole of the scaphoid to prevent minor conflicts between the scaphoid and trapezium and leaving a good mobility in all space planes.

### *C33—NEOPLASTIC DISEASES AND INFECTIONS 3*

### Combined treatment of pre-operative selective arterial embolization and surgical stabilization in patients with bone metastases of renal cancer

A. Di Francesco*[1], S. Flamini[1], C. Masciocchi[2], A.V. Giordano[2], A. Conchiglia[2]

[1]Ortopedia e Traumatologia, O.C. S. Salvatore, ASL1 L'Aquila (L'Aquila, IT);
[2]Dipartimento di Radiologia, Università degli Studi de L'Aquila (L'Aquila, IT)

**Introduction** The aim of the study is to report the experience of our Institution on combined treatment with selective arterial embolization of bone metastases from renal cell carcinoma and subsequent surgical stabilization.
**Materials and methods** From 2006 to 2011, 15 patients with renal cell carcinoma underwent embolization of 18 bone metastases. All patients had secondary lesions in the femur or tibia at high risk of pathologic fracture and were intended for prior surgical stabilization. The selective arterial embolization was performed in order to facilitate the surgery, reducing intra operative blood loss. The embolization was performed only after careful consideration of arteries and collateral vessels and the embolic agent was the N-2-butyl-cyanoacrylate (NBCA). Surgery was always performed within 3 days from the procedure and estimated intraoperative blood loss have been compared with those of 13 control cases evaluated retrospectively. Diaphyseal lesions were treated with nailing (10 cases), while those with periarticular plates with angular plates (5 cases). The evaluation parameters were: assessing the vascular supply to the lesion, blood loss and pain.
**Results** All embolizations have been completed with success. Il technical success was assessed by angiography overview, performed at the end of the procedure that in all cases showed, at the level lesion, a complete interruption of vascular or a reduction in the vascularity of more than 80 % compared with initial exam. In all cases there was a significant reduction of intraoperative blood loss compared to the control cases (mean blood loss 630 ml ± 400 ml *vs.* 4000 ml ± 2000 ml). The patients experienced a complete or partial response in 95.1 % of the cases handled by the combined treatment and in only 59.9 % of cases treated surgically. We achieved technical success in 100 %. The mean VAS increased from 6.5 to 2.1 with a reduction of 68 %. There were no major complications.
**Discussion** It is important to find procedures that allow an improvement of conditions of life of patients with renal cell carcinoma with bone repetitive processes.
**Conclusions** The selective arterial embolization of bone metastases of renal cell carcinoma, which in most cases are hypervascular, significantly reduces the intra-operative blood loss and a clear improvement in pain symptoms.

### *C34—KNEE 6*

### Patient matched TKA instrumentation Visionaire versus standard TKA: preliminary results

M. Galgani*[1], M. Tenucci[1], S. Vallini[1], S. Cozza[2], G. Calvosa[1]

[1]U.O. Ortopedia e Traumatologia (Volterra, IT);
[2]U.O. Diagnostica per Immagini (Volterra, IT)

**Introduction** The aim of this study is to compare new patient matched TKA cutting block (Visionaire, Smith & Nephew Inc., Memphis, Tenn) (VIS) with standard TKA (STD).
**Materials and methods** 40 knee osteoarthritis patients were enrolled and randomized (20 for VIS TKA 20 for STD TKA). Both groups performed pre-op imaging (X-rays), TKA VIS performed MRI following Visionaire protocol. Lower limb axis and ROM were evaluated pre-op. In both group we used, mid-vastus access, Genesis II Knee System. We compared surgical times. We evaluated post-op. lower limb axis with X-rays and compared day-time to performed a lower limb elevation, surgical incision length and discharge days.
**Results** Our results demonstrate that TKA VIS group had a significant reduction of surgery time and surgical skin incision, X-rays better component alignment. In post-op. TKA VIS patients demonstrated a faster functional recovery and a hospitalization time reduction.
**Discussion** With so far results we can demonstrate that with VIS system TKA can be performed in lower time less invasivity. We have achieved a post-op. faster functional recovery with a hospitalization time reduction.
**Conclusions** These results show that VIS TKA system is an interesting technique. We will need to support this study with a higher patient number and longer follow-up.

### Custom-fit cutting blocks for total knee replacement: comparison between CT and MRI/X-ray systems

A. Timoncini*[1], A. Ensini[1], A. Feliciangeli[1], F. Cenni[2], M. Busacca[3], S. Giannini[1]

[1]Clinica II, Istituto Ortopedico Rizzoli (Bologna, IT);
[2]Laboratorio Analisi del Movimento, Istituto Ortopedico Rizzoli (Bologna, IT);
[3]Radiologia Diagnostica ed Interventistica, Istituto Ortopedico Rizzoli (Bologna, IT)

**Introduction** In the last decade computer-assisted techniques have been developed to make Total Knee Replacement (TKR) safer and more accurate. Since 2009 Medacta International and Smith&Nephew have created two innovative methodologies that from CT or MR of the patient are able to create a preoperative surgery planning and build femoral and tibia custom-fit cutting blocks for the implant positioning. The purpose of our study is to evaluate and compare the accuracy of these two methodologies in terms of alignment of custom-fit cutting guides, bone cuts and prosthetic components with respect to the preoperative planning.
**Materials and methods** We enrolled 50 patients with symptomatic osteoarthritis of the knee for TKR: 25 were assigned to Myknee® system (Medacta-International, Castel San Pietro, CH) and 25 to Visionaire® system (Smith&Nephew, London, UK). The two systems included the acquisition and uploading of CT scans of the hip, knee and ankle (MyKnee®) or MRI of the knee and weight-bearing X-rays of lower limb (Visionaire®) to a dedicated website, the approval of the preoperative web planning and the production of the custom-fit cutting blocks for TKR. In each patient-specific planning frontal, sagittal and transverse alignments of prosthetic components were indicated. We recorded intraoperatively with surgical navigator the alignment of cutting-blocks and bone cuts, and postoperatively with radiographs and video-fluoroscopy (shape-matching) of prosthetic components. We have compared the values collected with those of the pre-operative planning and we have considered as outliers differences more than 3°. Statistical comparison between the two systems was performed.
**Results** The percentage of outliers in frontal alignments was 10 % for Myknee® and 12 % for Visionaire®, in sagittal 33 % for MyKnee® and 18 % for Visionaire®, and in transverse 17 % and 12 % for MyKnee® and for Visionaire® respectively. The comparison between the two systems was not statistically significant ($p > 0.05$).
**Discussion** Both systems showed good accuracy in terms of alignment of the prosthetic components, in particular it was statistically similiar to each other and greater respect to that provided by traditional technique. MyKnee® has proved less accurate in femoral sagittal alignments, Visionaire® in tibia sagittal alignments.
**Conclusions** The custom-fit cutting blocks made with CT or MRI-X-rays systems provided good accuracy and were equally preferable in TKR.

### Patellar instability: MPFL reconstruction versus TT transfer. Our experience and considerations

A. Russo*[1], V. Condello[2], V. Madonna[2], F. Cortese[2], V. Iacono[2], D. Screpis[3], C. Zorzi[2]

[1]Università Federico II Napoli (Naples, IT);
[2]Ospedale Sacro Cuore (Negrar, Verona, IT);
[3]Università degli Studi di Perugia (Perugia, IT)

**Introduction** The management of patellar dislocation syndrome has traditionally been difficult: there are no golden standard methods. Four principal anatomic factors have been identified that increase the risk for episodic patellar dislocations: trochlear dysplasia, patella alta, patellar tilt, and an excessive tibial tubercle-trochlear groove distance. However, it is known that medial patella-femoral ligament (MPFL) is damaged when the patella is dislocated. The surgical treatment is aimed at restoring the congruence of the patellofemoral articulation and correcting extensor mechanism malalignment. Current research suggests that MPFL reconstruction is an important surgical option in the treatment of lateral patella-femoral instability. Success after MPFL surgery depends on a detailed understanding of precise MPFL origin and insertion landmarks, compensation for trochlear deficiency, proper tensioning, and understanding when to add or substitute a tibial tubercle transfer. Tibial tubercle transfer is a useful adjunct to MPFL reconstruction in patients with an high TT-TG index, to correct the patellar index in patella alta, to add compensatory balance in the presence of trochlear dysplasia and to offload some patella-femoral articular lesions, or more frequently a combination of them.
**Materials and methods** We reviewed, retrospectively, 100 patients (120 knees) who had undergone surgery (between 2005–2008, with mean follow-up of 5 years) diagnosed with "habitual dislocation", "recurrent dislocation", "traumatic dislocation" and "unstable patella". The surgeons performed lateral release, vastus medialis advancement, MPFL plasty or reconstruction, tibial tubercle transfer and frequently a combination of them. All patients were evaluated by physical findings using Kujala's score, ROM and imaging.
**Results** The mean Kujala score improved significantly from 61.3 points preoperatively to 93.4 points at follow-up. No patient experienced patellar dislocation between surgery and final examination, although 10 patients (13 knees) had experienced the subluxation sensation and the apprehension signs remained. The main ROM was from $0 \pm 5°$ to $145 \pm 5°$. The worst results were occurred in association with cartilage lesions.
**Discussion** Most of the time failures resulting from poor preoperative assessment. Instead, in case of correct diagnosis all surgical treatments have proven successful, both individually and in combination.
**Conclusions** From the review of cases highlights the importance of the preoperative study to adapt the right treatment to the individual patient's pattern. At last, when cartilage defects are associated with patellofemoral malalignment, we recommended an anteromedial TT transfer.

### Reliability of tibial crest for coronal extramedullary alignment in total knee arthroplasty

P. Sessa*, A. Della Rocca, F.R. Ripani, F. Gabriele, G. Cinotti

Clinica Ortopedica, Università La Sapienza (Rome, IT)

**Introduction** Several anatomical landmarks are currently used for coronal alignment of tibial extramedullary guide in total knee arthroplasty (TKA). However, the reliability of most of these landmarks remains to be elucidated. This study was aimed at analysing whether the tibial crest may be used as bony landmark for coronal alignment in TKA.

**Materials and methods** Forty-eight dried cadaveric tibiae were analysed. The tibial mechanical axis was firstly identified by calculating the middle of the proximal and distal tibial joint surface. Both the proximal and distal extremities of the mechanical axis were translated to the anterior tibial cortex to obtain an anterior tibial longitudinal axis (ATLA) overlapping the coronal mechanical axis. The intersection points between the ATLA and the tibial crest and between the former and the tibial tuberosity were recorded.

**Results** An ATLA passing through the medial 1/3 of the tibial tuberosity, was found to intersect the tibial crest in 55.31 % of cases (in 36.17 % in 2 points, in 17.02 % in 1 point). An ATLA passing through the middle of tibial tuberosity was found to intersect the tibial crest in 100 % of cases (in 78.7 %, in 2 points, in 23.4 % in one). An ATLA passing through the medial border of the tibial tuberosity, intersected the tibial crest in only 13 % of cases.

**Conclusions** Results of this study indicated that the tibial crest is aligned in 100 % of cases with an ATLA passing through the anterior projection of middle of the talar dome and the middle of the tibial tuberosity. However, the anterior projection of the tibial mechanical axis, perpendicular to the transepicondylar femoral axis, was found to intersect the medial 1/3 of the tibial tuberosity in most of the subjects. As a result, if one considers the medial 1/3 of the tibial tuberosity as the proximal projection of the coronal mechanical axis, the tibial crest axis is tilted about 3°–4° laterally compared with the coronal mechanical axis. As a result, the tibial component may be placed with 3°–4° of valgus when the tibial crest is used as bony landmark for coronal alignment.

### All-inside anatomical ACL reconstruction: surgical technique and preliminary results

R. Buda*, A. Ruffilli, M. Cavallo, A. Parma, G. Pagliazzi, F. Luiso, S. Giannini

II Clinica Ortopedica e Traumatologica, Istituto Ortopedico Rizzoli (Bologna, IT)

**Introduction** Anterior cruciate ligament (ACL) reconstruction has become a common procedure, with several surgical techniques described. Recently technical improvements led to the development of minimally invasive techniques capable to obtain anatomic ACL reconstruction. Aim of this study is to describe the preliminary clinical results obtained with an anatomic, single bundle all-inside technique using triplicated or quadruplicated autologous semitendinosus tendon.

**Materials and methods** 19 male athletes with unilateral ACL injury were enrolled in the study. The technique encompassed the unconstrained creation of an half tunnel both on the tibial and on the femoral side using guide pins of 3.5 mm in diameter that became retrograde drills. Femoral and tibial fixation was achieved with second-generation cortical suspensory fixation devices with adjustable graft loop length. The patients were followed up with clinical examination, International Knee Documentation Committee (IKDC) scoring system and KT-1000 arthrometer with a minimum 12 month follow-up duration.

**Results** No intra-operative nor post-operative complications were observed. Normal R.O.M. was observed in all patients at follow-up. Significant improvement was seen on Lachman test and Pivot-Shift test between pre-operative and last follow-up. KT-1000 side-to-side difference averaged 0.7 ± 0.2 mm. Average IKDC subjective score at follow-up was 97.1 ± 1.4, IKDC objective score was normal in 18 cases (A) and nearly normal in 1 cases (B). All patients resumed sport activity at pre-injury level.

**Discussion** The use of sockets instead of tunnels associated to the use of second generation cortical suspensory devices permit to reduce the bone loss and guarantee the best bone-graft contact enhancing the ligamentization process of the graft. The use of one single tendon, triplicated or quadruplicated, represents an optimal solution for knee biomechanics. Major limitations of this study are the short follow-up and the lack of prospective randomized studies comparing this technique with earlier standardized procedures.

**Conclusions** The described technique showed to be minimally invasive and effective in restoring knee stability.

### The Nexgen total knee replacement: ten-year results

M. Vasso*, S. Cerciello, A. Felici, A. Schiavone Panni

Università del Molise (Campobasso, IT)

**Introduction** The long-term success of total knee replacement is multifactorial including factors relating to the patient, the operation and the implant. Polyethylene wear remains the main cause of failure and revision of total knee arthroplasty after 10 years. The Nexgen system is a third-generation prosthetic system, designed to address the following problems with previous implants: polyethylene wear, osteolysis, patella-femoral problems.

**Materials and methods** From 1998 to 2001, 180 primary Nexgen total knee arthroplasties were implanted at our institution. Of these, 150 (142 patients) had a follow-up over 10 years. 132 PS and 18 CR, 111 fixed and 39 mobile arthroplasties were implanted. At surgery, mean age of the patients was 71.2 years. Primary diagnosis was osteoarthritis in 133 patients, rheumatoid arthritis in 8, avascular necrosis in 5, other in 4. Patella was resurfaced in 44 knees. All data were prospectively collected. Patients were assessed using the IKS knee and function scores, preoperatively and postoperatively after 3, 6, and 12 months, and yearly thereafter. Radiographic evaluation was completed for all knees. Failure was defined as loosening or revision of any component for any reason.

**Results** IKS knee and function scores improved from 42 ± 4 and 48 ± 3 preoperatively to 82 ± 5 to 84 ± 3 to the latest follow-up ($p < 0.0001$). Average flexion increased from 98° ± 3° preoperatively to 123° ± 5° at the latest follow-up ($p < 0.001$). IKS knee and function scores were similar in PS group and in CR group, such as implant survivorship. We found no significant differences neither in post-operative clinical, functional, radiographic results, nor in the polyethylene wear and osteolysis in fixed and mobile bearing groups. Seven (4.6 %) failures were reported.

**Discussion** In our series, a 10-years survivorship of 95.4 % was achieved. This confirms the excellent survival rates of primary knee replacements. The Nexgen system simplify surgeries, save operating time, reduce costs, match prosthetic components to specific patient demand levels and gender. Reduced width and thickness of the femoral flange and deepened patellar groove relieve pressure on the extensor mechanism reducing morbidity, lateral retinacular release rates and premature wear.

**Conclusions** These results show that with appropriate patient selection and meticulous attention to surgical technique, excellent clinical and radiographic long-terms results can be achieved thanks to modern total knee implants.

### *C35—BIOTECHNOLOGIES*

### Use of PRP in the arthrodesis of the lumbar spine: medium-term results

A. Zachos*, F. Muresu, A. Ruggiu, C. Meloni, C. Doria

U.O.C Ortopedia e Traumatologia (Oristano, IT)

**Introduction** It is well known that the platelet-rich plasma (PRP), is a safe and convenient source of growth factors, which can stimulate other cells to regenerate a tissue. This concentrate contains condensed nucleated cells of bone marrow and platelets condensed. This simultaneous concentration of platelets and bone marrow cells has great potential as a source of materials for regenerative medicine.
**Materials and methods** At the Orthopaedic Clinic of Sassari and the Division of Orthopaedics and Traumatology of Oristano, from June 2008 to January of 2011, 92 spinal arthrodeses were performed. In one half of the patients the concentrate of bone marrow and platelets condensed were applied, derived from the iliac crest by the method ''marrow stem''. The arthrodeses were of two types: circumferential and postero-lateral. The preoperative diagnosis was: (1) degenerative disc disease with radiculopathy (42 cases) and coexisted a condition of instability; (2) degenerative spondylolisthesis (32 cases) and; (3) isthmic spondylolisthesis (18 cases). The following levels were treated: L2-L3 in 12 cases, L3-L4 in 27 cases, L4-L5 in 29 cases and L5-S1 in 24 cases. The evaluation criteria took into account the clinical and radiographic results with the help of evaluation forms for pre-and post-operative VAS (Visual Analogic Scale) from 0 to 10 points and ODI (Oswestry Disability Index) from 0 to 100 %.
**Results** Patients were evaluated at a mean follow-up period of 12 months (range 1–24 months). In any case we reported intraoperative complications. In one case we observed the subsidence of the cage in the lower vertebral body and in one case we observed the placement of the cage is too back that required surgical revision at a distance. The average score of postoperative VAS was 2.9 compared to the pre-operative 8.2. The postoperative average score of ODI was 36.8 ODI % against 64.2 % preoperatively. The analysis of postoperative radiographs taken at a distance of 3, 6 and 12 and 24 months after surgery showed signs of fusion much earlier in patients where was applied the concentrate.
**Discussion** It is well known the use of PRP in spinal surgery.
**Conclusions** In all cases where the concentrate was used, we had excellent functional arthrodesis with excellent results in a time shorter than the vertebral fusions where the PRP was not used.

### Study protocol for osteoblastogenesis modulation drugs in experimental fractures of murine models

C. Ruosi*[1], F. Querques[2], F. Granata[1], A. Barbato[1], S. Lupoli[1], G. Colella[1], M. Sposato[3], L. Pastore[2]

[1]Dipartimento di Ortopedia e Traumatologia, Università degli Studi di Napoli “Federico II” (Naples, IT);
[2]Dipartimento di Biochimica e Biotecnologie Mediche e CEINGE-Biotecnologie Avanzate, Università degli Studi di Napoli “Federico II”(Naples, IT);
[3]CEINGE-Biotecnologie Avanzate (Naples, IT)

**Introduction** Bone fracture therapies are generally studied using large size animal models; but these models are expensive and do not allows the study of the effect of genetic alterations on the bone repair. The aim of this study is to investigate the effects of new drugs on the osteoblastic activity in a murin model of bone fracture. For this purpose experimental femoral fractures are made in C57bL/6 mice. With radiological and histological procedures will be evaluated the effect of drugs administrated per os or in the fracture site.
**Materials and methods** Female C57bL/6 mice are used for this experiment. All animals are between 6 and 8 weeks old. After anaesthesia with intramuscular injection of ketamine hydrochloride and acetylpromazine a 1-cm right medial parapatellar incision is made. The longitudinal fibers of the quadriceps mechanism are divided and the patella is dislocated laterally to expose the femoral condyles. A sterile 25-gauge needle is introduced into the intramedullary canal and advanced in a retrograde fashion up the shaft of the femur to the level of the greater trochanter. Using dissection, the diaphysis is exposed. A small snipper is used to fracture the middiaphysis of the femur. The soft tissues are closed. The left femur is left unfractured. Animals are allowed free, unrestricted weight bearing after recovery from anaesthesia. A group of mice (n = 5) is treated with drugs that was effective in vitro. Animals are examined with DEXA scanning to evaluate the bone mineralization in the fracture site. In the end the fractures are valuated with DEXA, micro CT and, after the sacrifice, histological analysis.
**Results** A group of mice (n = 5) is treated with drugs that was effective in vitro. Animals are examined with DEXA scanning to evaluate the bone mineralization in the fracture site. In the end the fractures are valuated with DEXA, micro CT and, after the sacrifice, histological analysis.
**Discussion** The aim of this procedure is to study effects of new drugs identified with high-throughput screening on fracture healing.
**Conclusions** It is analyzed how osteoblastogenesis can be manipulated with modulation of specific proteins that have a role in the cellular proliferation of the callus formation. The aim of this study is to obtain preclinical data of efficacy in order to evaluate the possible clinic use of these drugs.

### Adult adipose mesenchymal stem cell implantation for one step knee chondral defects repair: a new technique

F.V. Sciarretta*, C. Ascani

Clinica Nostra Signora della Mercede (Rome, IT)

**Introduction** The management of articular osteochondral defects, whether from trauma or, always more frequently, due to population demographic changes, from degenerative disease, continues to be a significant challenge for orthopaedic surgeons. Stem cell therapy has become one of the main “one step surgery” choices. Mesenchymal stem cells represent a potential source of cells for cell-based cartilage repair due to their ability to self-renew and undergo multi-lineage differentiation and their use has the advantage to avoid primary surgery for cartilage biopsy and subsequent chondrocytes cultivation. Adult MSCs are mainly isolated from bone marrow or fat tissue. In this paper we discuss the use of adult adipose tissue-derived stromal cells (ADSCs) for the repair of knee focal full thickness chondral defects by the use of an improved technique (MyStemTM) for isolating adult viable mesenchymal stem cells from lipoaspirate within less than 30 min.
**Materials and methods** Once the chondral defect has been arthroscopically identified and debrided, in local anesthesia, we extract, by a simple, minimally invasive method of liposuction aspirate, with a specifically created adipose tissue biopsy needle, the marrow tissue from adult adipose tissue of the abdomen. The vacuum syringe with the few ml of liposuction tissue is, bedside in the OR room, connected to the collection bag of pre-treatment, where the infuse content is mechanically filtered. Then the stromal-vascular fraction of cells is separated from the mature lipid-laden adipocytes and the water cellfree component by centrifugation according to a specific centrifugation protocol for 10 min. This fraction contains ADSCs in a large number, with yields of approximately 250,000 cells per gram of tissue. Then, adding fibrin glue to the concentrated ADSCc, we obtain a sticky clot ready to be easily implanted in the osteochondral prepared defect.
**Results** We have prospectively followed the grade 3 and 4 chondral defects of the knee treated by ADSCc implantation with or without the use of a collagen membrane scaffold. All the patients have been treated by the same surgeon and have followed the same post-operative regimen. Patients have, at short term follow-up, showed improvements in all scores and no adverse reaction has been noted.
**Discussion** This proposed procedure is simple, quick and low cost. Specifically, it has the advantage of not requiring harvesting of cells from the joint surface, and its associated donor site morbidity. The adipose tissue lipoaspirate procedure is well known, simple and low-risk by the use of the specific blunt-tipped biopsy needle. The direct culture in the OR of the adipose mechanically separated layer of cells, without the need to perform collagenase lysis, makes it a quick procedure, easy and safe to perform in the OR, and perfectly adapting to the timing requested for a single step chondral defect repair procedure.
**Conclusions** As already described by many authors, one step patient-side surgery is certainly becoming the technique of choice for chondral and osteochondral defects repair. This single-step ADSCc implantation procedure reduces time, costs and is less invasive to the patient, but although promising, needs more patients and longer follow-ups.

### Early effects of pulsed electromagnetic fields on human osteoblasts and mesenchymal stem cells

V. Sollazzo*[1], A. Palmieri[2], A. Girardi[2], F. Carinci[3]

[1]Clinica Ortopedica, Università di Ferrara (Ferrara, IT);
[2]Laboratorio Interdipartimentale di Chirurgia Maxillofacciale ed Ortopedia, Università di Ferrara (Ferrara, IT);
[3]Istituto di Chirurgia Maxillofacciale, Università di Ferrara (Ferrara, IT)

**Introduction** Pulsed Electromagnetic Fields (PEMFs) are commonly used in the clinical practice to treat several pathologies like osteotomies, avascular necrosis of the femoral head and bone grafts. The sequences of events by which electromagnetic stimulation can lead its effects on bone healing are not completely understood. In order to get more information on the mechanism of action of PEMFs, we asked whether (a) PEMFs are able to induce changes in the production of growth factors in human osteoblasts and in human mesenchymal stem cells, (b) the production of growth factors induced by PEMFs vary at different time points (12 and 24 h), (c) PEMFs induce a different production of growth factors in cells at different stages of differentiation like human mesenchymal stem cells and human osteoblasts.
**Materials and methods** Therefore, we exposed normal human osteoblasts and mesenchymal stem cells cultures to PEMFs and then we evaluated the expression of 84 growth factors through rtPCR analysis.
**Results** Twenty seven genes out of the 84 studied were significantly over and down expressed and fourteen of those genes up or down regulated are related to bone formation or inflammation.
**Discussion** Our data demonstrate that PEMFs exert their effects by activating or suppressing a large number of growth factors simultaneously with different functions and that this variation of expression varies depending on time and depending on the different stage of differentiation of the cell.
**Conclusions** In this study, we demonstrated that PEMFs are able to improve cell differentiation and to promote bone healing.

### *C36—HAND AND WRIST 2*

### Ulnar osteotomy in the treatment of ulno-carpal impaction: 10 cases

M. Zanlungo*, F. Nasi, E. Finardi, F. Caracciolo

Istituto di Cura Città di Pavia (Pavia, IT)

**Introduction** There is a subjective variability of ulna length valuated with radio-ulnar index ± 2 mm or Hulten variant. Distal radius fractures are most likely to give long-term symptoms when malunion involves intra-articular incongruity, shortening or residual dorsal angulation. Shortening results in malalignment D.R.U.J and in ulno-lunate impaction syndrome. Impaction syndrome is also frequently in Madelung deformity.
**Materials and methods** We have operated 10 symptomatic patients, 7 women and 3 men, average 32 to 72 years old; 7 radius distal fractures outcome and 3 Madelung. Osteotomy was ever oblique and ostheosyntesis done with 7 holes plate and optional screw across osteotomy. Actual plates that supply instruments drive to exact double osteotomy. Angular stability screws give a first stability who avoids longer brace immobilization. These plates must be applied on the anterior ulnar surface. The opening wedge, in the past dorsal into the FUC and EUC interval, now is anterior with mobilization of the FUC; ulnar diaphysis is discovered with periosteal preservation; only in the osteotomy surface periosteous was removed. The osteotomy, in the past at 6 cm from the ulnar styloid, now is at the distance point by the guide applied on the plate. The compression plate is now with 6–7 holes, sufficiently resistant to support immediate charge. The screw 3-5 mm must be at angular stability with eventually inter-fragmentar screw. The modern Acumed plates permits a shortness until 10 mm without plate strain instrument. We used Acumed plates in the last 3 cases. In the other 7 cases we used Syntes 6 hole-plates.
**Results** All cases consolidated with complete remission of pain; in 1 case we had a consolidation retard resolved with prolonged brace immobilization and "pulsated aging therapy".
**Discussion** In the symptomatic malunion of distal radius we prefer firstly correction of radius deformity with length restoration. If the radius orientation is acceptable, or in case of insufficient correction, we perform ulna shortening.
**Conclusions** Ulna shortening with diaphisar osteothomy is a very good intervention to eliminate positive ulnar variants post-traumatic (radius distal fractures outcomes) or epiphysiolysis, if the DRUJ is congruent: ATC study must clear-up. Ulnar diaphysis shortening decompress radio-ulnar joint and stabilize fibro-cartilage and strain palmar and dorsal ligaments. Modern plates of ulna simplify operating and give an excellent primary stability.

### Early mobilization without protection in metacarpal and phalangeal fractures treated with internal fixation

D. Bazzoni*[1], M. Seves[2], M. Ramella[3,4], M. Merlo[1], A. Mazzucco[1]

[1]Ospedale di Circolo (Busto Arsizio, Varese, IT);
[2]Studio Ramella Seves (Busto Arsizio, Varese, IT);
[3]UOC Riabilitazione della Mano, IRCCS Multimedica (Castellanza, IT);
[4]UOC Riabilitazione della Mano, IRCCS Multimedica, Ospedale San Giuseppe (Milan, IT)

**Introduction** Modern and gold standard treatment of hand fractures contemplates, according to many authors, perfect anatomic reduction, early mobilization and early recovery of function. Except for the thumb, where we used for biomechanics reason a removable splint, in the last 3 years, we always aimed at stable internal fixation and early motion without protection for treating metacarpal and phalangeal fractures.

**Materials and methods** From September 2009 to nowadays, we have treated 25 fractures, 15 metacarpal and 10 phalangeal fractures. The inclusion criteria were: same surgeon, internal fixation, 2 therapists applying the same rehabilitation program, therapy beginning maximum 15 days after surgery. Internal fixation was always performed with plate and screws or only screws. For the metacarpals we used a dorsal surgical approach moving to the side extensor tendons; for the P1 and P2 dorsal surgical approach splitting extensor tendons: in this case, closure is performed in layers applying gel to avoid adhesions. Surgery aimed at obtaining an absolute stable internal fixation to allow early sheltered motion. Patients were discharged from Hospital with a simple dressing; indications are early autonomous mobilization and precise pain therapy; 2 days after operation, we lightened the dressing, checked the surgical wound and sent the patient to the therapist to begin motion. We recommended the patient to use the hand avoiding strength. One month after operation, after clinical and radiological evaluation, we allowed gradual strengthening exercises.

**Results** All patients regained complete ROM. Mean time of discharging from rehabilitation program was 35 days. We observed 1 case of plate breakage with secondary healing of the fracture, without reoperation or stopping of the rehabilitation. We had not complications as sepsis, hardware-related pain or complex regional pain syndrome. No patients needed tenolysis o re-operation for joint stiffness.

**Discussion** Hand surgery is a functional surgery. Patients of all ages, if affected by fractures of the hand, increasingly ask for a early recovery of function and anatomic restoration. These requests are the objectives of the surgeon, who has to improve techniques of internal fixation highly stable and minimally invasive, dispense effective pain therapy and cooperate closely with the Therapist.

**Conclusions** Early mobilization without protection ensures, in our experience, an early recovery of hand function; the patients return faster to manual and working activities. There is no indication in uncooperative patients and children.

### External fixation in traumatology of the hand: our experience at 5-year follow-up and literature review

A. Tomarchio*, G. Bruno, E. Marchese, D. Ghargozloo

Sezione di Ortopedia e Traumatologia, Dipartimento delle Specialità Medico Chirurgiche, Università degli Studi di Catania, Azienda Ospedaliera Universitaria Policlinico Vittorio Emanuele (Catania, IT)

**Introduction** External fixation is a useful and adaptable tool for the treatment of hand fractures, especially when associated with soft tissue injuries and loss of bone. However this system has limits: the fracture fragments may not be controllable and can be difficult to build the system for the little space available.

**Materials and methods** We report our experience in the use of Orthofix Pennig Minifixator (OPM) in metacarpals and phalangeal fractures. Were treated surgically, from May 2007 to February 2012 with mean follow-up 32 months, 47 patients (38 males and 9 females) who had a mean age of 34.2 years (16-81): 28 cases of metacarpals fractures, 21 of phalanges. More involved was the thumb metacarpal base (15 cases) always treated with OPM and screw; little finger proximal phalanx (9 cases) often associated with ring finger middle phalanx fractures (5 cases). In two cases we have seen associated fractures of metacarpals (index and middle–middle and ring) treated simultaneously. Eleven cases of open fractures.

**Results** Outpatients clinic follow-up and surgical wound dressing were performed every 7 days until OPM's removal. We performed X-rays in A&E Department, post op., after 30 days and a last check at maximum follow-up. The OPM was removed after the healing of fracture in dressing clinic on average at 42 days. We evaluated the range of movement, strength and any presence of malrotations. The results were good: we, however, found pins failure in two cases and superficial skin infections in three cases.

**Discussion** The evaluation of literature confirms a key role in complex trauma of the hand, while the use of external fixation has not a main indication in "simple" fractures of metacarpal and phalanx. The use of this depends on the preferred techniques by the authors, there's not a unique indication to this method in literature. In particular we propose the use of OPM associated with Synthes HCS screw in thumb metacarpal base fractures.

**Conclusions** External fixation in traumatology of the hand is an efficient system that allows fracture healing through callus formation, avoiding exposure of the fracture. The possible association of minimal internal fixation is not considered a contraindication. The functional recovery depends on the reduction of the fracture, by the correct construction of the external fixator and early mobilization of the affected segment. The use on middle and ring metacarpal is non comfortable, even if it represents a good alternative to prolonged immobilization.

### Long-term follow-up of a false aneurysm of the palmar arch

M.C. Sbernardori, P. Zedde*, A. Melis, M. Cavazzuti, A. Santandrea, P. Tranquilli Leali

Clinica Ortopedica Sassari (Sassari, IT)

**Introduction** False aneurysm of the palmar arch is a very rare clinical condition and has been reported infrequently; it is a vascular dilatation developed from an arterial hematoma that is secondary to an opening into the arterial wall.

**Materials and methods** A 29-year-old male was seen 10 days after a stab wound in the first interthenar space of the right hand followed by a progressive formation of a pulsating lump. Arteriography and ultrasonographic examination confirmed the presence of a false aneurysm of the metacarpal artery of the thumb at the detachment from the palmar arch.

**Results** 15 days after the surgical suture of the involved artery, after an initial disappearance of the mass, ultrasonographic examination documented a recurrence. Ultrasound follow-up at 30, 45 and 60 days after surgery highlighted a progressive organization of the aneurysm sac and its progressive natural closure, simultaneously with the resumption of functional activity of the hand. The aneurysm sac was re-evaluated 24 months after surgery.

**Discussion** Nowadays the treatment of the false aneurism is controversial: most of the authors suggest surgical repair of the sac to prevent possible embolism and digital ischemia. However, the limited cases described in the literature do not allow certain conclusions.

**Conclusions** Due to the development of the case with a 24-month follow-up, the authors describe the natural evolution of the lesion with its closure and analyze the therapeutic implication.

### Diagnostic flow-chart for acute scapholunate ligament sprain

M. Rosati*, A. Poggetti, P. Battistini, L. Dolfi, M. Lisanti

I Clinica Ortopedica Universitaria (Pisa, IT)

**Introduction** The diagnosis of rupture of the scapholunate ligament still remains a challenge for orthopaedics and surgeons of the hand.

The complexity of the carpal biomechanic, the poor knowledge of the clinical tests and unsuitable management of diagnostic tools are all guilty for an incorrect diagnosis. The aim of this study is to set up a suitable diagnostic approach in the emergency room.

**Materials and methods** From 2008 to 2011, 44 acute lesions of scapholunate ligament were diagnosed at the 1st Orthopaedic Dept. of the University of Pisa. Among these 11 cases were diagnosed in the emergency room immediately after the trauma (2 cases of dissociation of predynamic SLIL and 9 cases of dynamic dissociation), the other 33 cases were developed within 2 weeks. Pointing our attention on the 11 cases of immediate diagnosis we observed 8 male and 3 female, average age 38 years (min. 17, max. 56), 7 right and 4 left wrist, 8 sport injuries and 3 accidental falls. In order to diagnose anamnesis of the trauma and specific clinical tests were used (scaphoid shift test, scapholunate ballotement test) and we carried out readiographic static examination, comparative X-rays, dynamic imaging under fluoroscopy.

**Results** All the patients felt pain at the dorsal surface of the wrist, and gained pain during clinical tests. We observed 9 positive scaphoid shift test, whereas 6 cases during scapholunate ballotement test. On plain X-rays we never observed static deformities such as Terry Thomas sign. On the contrary, stress test under fluoroscopy was positive in 9 cases. In 2 cases we needed arthro-MRI to make diagnosis.

**Discussion** On our experience and in accordance with more recent literature, we felt that the clinical tests are a key stonein order to suppose an acute lesion of scapholunate ligament, but at the same time they are not specific. The dynamic X-rays examination however made us able to put diagnosis of acute scapholunate ligament rupture. If in doubt arthro-MRI can be useful, but only if performed by experienced radiologists.

**Conclusions** In our experience we believe that a systematic approach to the trauma of the wrist is basic to reduce the number of missing diagnosis of scapholunate rupture. If the diagnosis is prompt, the surgical treatment is easier and gives better results.

### The biological arthroplasty in surgical treatment of rhizoarthrosis: proposal for a technical change

N. Galvano*, M. Lauria, M.C. Agnello, S. Moscadini

Policlinico Universitario di Palermo (Palermo, IT)

**Introduction** The authors in this paper, after a 4 year, show that, to date the biological arthroplasty is the treatment of choice, if not the only feasible treatment in rhizoarthrosis.

**Materials and methods** Between 2007 and 2010 we submitted 23 patients, 9 women and 14 men to intervention and emitrapezectomia arthroplasty using biological emitendine transposition of APL around the FCR. In this treatment was followed by immobilization in a plaster splint for three weeks.

**Results** In all cases occurred in understanding the healing marked reduction or abolition of pain symptoms, in correcting anatomical deformities and in the excellent restore mobility of the radius. In 15 % of cases occurred the occurrence of a tendonitis of the flexor carpi radialis which resolved with medical and physical therapy in 12 % of cases.

**Discussion** With this variation in technique, there were no collapses of the radius determined by the total removal of the radius. All cases were assessed at 6 months and 1 year after surgery using VAS and MAJO CLINIC. Variant of technical confidence, then only in cases of trapezio-metacarpal joint osteoarthritis isolated to prevent the collapse of the first ray.

**Conclusions** Against arthrodesis, the emitrapezectomia associated with biological arthroplasty has the conservation of a greater mobility of the first beam in the absence of functional overload of the trapezium scaphoid. Our technique and in particular the partial removal of the trapezium, on the one hand leaves a new articulation, whilst also preventing the collapse of the I present in the MTC trapezectomia total without affecting the clinical results and functional.

### *C37—NEOPLASTIC DISEASES AND INFECTIONS 4*

### The surgical site infection on a traumatized patient: correlation among age, diabetes, smoke and operating risk

L. Fisichella[1], D. Fenga[1], M. Maio[1], A. Laudati[2], V. De Santis[3], M.A. Rosa*[1]

[1]Scuola di Specializzazione in Ortopedia e Traumatologia, Dipartimento Specialità Chirurgiche, Sezione di Ortopedia e Traumatologia, Università degli Studi di Messina (Messina, IT);
[2]U.O.S. Chirurgia Artroscopica, ASL RME (Rome, IT);
[3]Dipartimento Specialità Ortopediche, Università Cattolica (Rome, IT)

**Introduction** Surgical site infection is a typical event after orthopaedic-surgery operations and it can be associated with increase of morbidity and social cost. The accurate identification of risk factors is essential to elaborate strategies to prevent these potentially devastating infections. The Authors have conducted a study to determine possible risk factors of the surgical site infection.

**Materials and methods** From October 2009 and December 2011, 84 patients with a superficial and/or deep surgical site infection were identified and compared with 203 uninfected patients (control group), taken out from a series of 486 patients. Analysed risk factors were: diabetes, BMI $> 30$, ASA Score 3 or 4, smoke and age.

**Results** The most performed operations were the knee and hip prostheses ($n = 32$, 11.14 %) and open reduction of fractures ($n = 178$, 62.02 %), on the 287 examined patients. The most common identified pathogen was *Staphylococcus aureus* ($n = 63.75$ %). The analyses (preoperatively and after the operation) on infected patients, compared to the control-group (odds ratio = 8,7), showed significantly high serum levels of glucose. It has been noticed an important incidence of infection inpatients smoking from more than 20 years, (67 patients, 79.7 %). Remaining variables tests (BMI, odds ratio = 2.21; ASA Score, odds ratio = 1.3), had no significant variation in the two groups (research and control). In addition, Authors noticed a correlation between $> 65$ years old patients and infection of surgical site (61 patients, 72.6 %).

**Discussion** The purpose of this research was to collect exhaustive elements concerning the potential risk factors of infection in orthopaedic-surgery. Results among diabetes, smoke and age as risk factors are correlated with development of an infection at the surgical site; while it is irrelevant the study of the ASA Score and BMI variables (contrary to what it is reported in literature).

**Conclusions** In authors' opinion a proper antibiotic treatment, the right glycemic control (achievable only with a careful multidisciplinary management) and a good acquiescence of the patient could assure a better management of the post operation results.

### Concentration and inhibitory activity of gentamicin and vancomycin in surgical drains placed in patients undergoing revision surgery with application of antibiotic-loaded spacer in prosthetic hip and knee infections

B. Magnan*, E. Bertazzoni Minelli, A. Benini, E. Samaila, M. Bondi, P. Bartolozzi

Policlinico Borgo Roma (Verona, IT)

**Introduction** The aim of this study was to asses the concentration of gentamicin and vancomycin in the first 24 h from surgical drains placed in patients undergone revision surgery for prosthetic hip and knee infections with implantation of a pre-formed antibiotic-loaded polymethylmethacrylate (PMMA) spacer, testing the inhibitory activity toward multiresistant microorganisms.

**Materials and methods** 12 patients were treated for infected hip and knee arthroplasty revision with "two-stage" procedure and application of a pre-formed antibiotic-loaded polymethylmethacrylate (PMMA) spacer supplemented with gentamicin (Tecres, Sommacampagna, Italy). Different vancomycin concentrations (1.25 %, 2.5 and 5 %) were mixed with PMMA during surgery. The surgical drains were collected at 1, 4 and 24 h post-operatively. The antibiotics concentration was determined by immunoassay in polarized light (TDx, Abbott). The title of bactericidal drainage was estimated to methicillin-resistant *Staphylococcus aureus* (MRSA) and *Streptococci* (CoNS).

**Results** The gentamicin and vancomycin release from the PMMA in the site of infection was found to be rapid and effective with high concentration in the first 24 h post-operatively. The kinetics of gentamicin and vancomycin release were similar, with a high inhibitory activity in the infection' site. The inhibitory activity of the samples was variable and related to the concentrations of antibiotic and to the tested microbial agents.

**Discussion** Gentamicin and vancomycin are released from the pre-formed antibiotic-loaded spacers with bactericidal concentrations and their use in association demonstrates a strong inhibitory effect towards MRSA and CoNS.

**Conclusions** The pre-formed antibiotic-loaded spacers appear to be an effective method for the release of antibiotics in the infection' site, adding an antimicrobial biological activity to the support of joint function at the intermediate stage, in two-stage treatment, facilitating the subsequent prosthetic re-implantation.

### Functional reconstruction around the knee with megaprosthesis

G. Caff*, D. Campanacci, F. Frenos, N. Mondanelli, G. Beltrami, G. Scoccianti, R. Capanna

Ortopedia Oncologica e Chirurgia Ricostruttiva, Ospedale Careggi, CTO (Florence, IT)

**Introduction** Extensive bone loss around knee may be due to bone tumor resections, conventional prostheses failure and posttraumatic sequelae. Distal femoral and proximal tibial defects can be effectively reconstructed by modular prostheses but in selected cases the use of an allograft-prosthesis composite (APC) may provide a functional advantage. Objective of the present study was to review our series of functional prosthetic reconstructions around knee (with and without allograft) with the aim to evaluate morbidity and implant survival.

**Materials and methods** From 2001, 194 patients (90 F, 103 M) were treated at authors' Institution with modular prosthetic reconstruction of the knee joint after resection of a malignant bone tumour in 142 cases, benign bone tumour in 36 cases and in bone loss due to non oncologic conditions in 16 cases. Megasystem C, Waldemar Link, was used in all reconstructions. A distal femur prosthesis was implanted in 135 cases (in one case a distal femur prosthesis was associated to an APC of proximal tibia) while a proximal tibia reconstruction was performed in 45 cases. Proximal tibial replacement was done with modular prosthesis in 23 cases and with APC in 22 cases. In 14 cases, due to the extension of the tumour into the joint space, an extra-articular resection was performed, with functional reconstruction by an APC with complete allogenic extensor apparatus.

**Results** At an average follow-up of 30 months, 54 major complications in 35 patients (18 %) were observed and 44 prosthetic surgical revisions were performed. Deep infection was observed in 20 cases (10.3 %). Eighteen prosthetic mechanical complications were seen (morse taper failure in 11 cases, articular joint failure in 5 cases, disassembling in 2 cases). Aseptic loosening occurred in 2 cases requiring surgical revision with a total femur prosthesis in 1 case. Two patients developed a wound dehiscence treated with a rotational flap. Patellar tendon detachment was seen in 7 cases, requiring surgical revision in 5 cases. Two traumatic patella's fractures and 1 traumatic periproshetic fracture were observed. Local recurrence occurred in 12 cases and surgical treatment was an amputation in 6 cases. Among the evaluable patients with at least a 12 months follow-up, 72 % showed satisfactory functional results (excellent or good following MSTS functional evaluation).

**Discussion** In our experience, Megasystem C showed to be an effective tool in articular reconstruction of segmental bone loss around knee. The conventional modular assembling was the preferred choice in distal femur. In proximal tibia reconstructions, conventional modular implants showed a higher incidence of infections and lower functional score than APC assembling.

**Conclusions** After extra-articular knee resection, an effective functional reconstruction was achieved combining the conventional modular assembling of distal femur to an APC of the proximal tibia with allogenic extensor apparatus.
